# Correlated x-ray fluorescence and ptychographic nano-tomography on Rembrandt’s *The Night Watch* reveals unknown lead “layer”

**DOI:** 10.1126/sciadv.adj9394

**Published:** 2023-12-15

**Authors:** Fréderique T.H. Broers, Ige Verslype, Koen W. Bossers, Frederik Vanmeert, Victor Gonzalez, Jan Garrevoet, Annelies van Loon, Esther van Duijn, Anna Krekeler, Nouchka De Keyser, Ilse Steeman, Petria Noble, Koen Janssens, Florian Meirer, Katrien Keune

**Affiliations:** ^1^ Science Department, Conservation & Science, Scientific Research, Rijksmuseum, Hobbemastraat 22, 1071 ZC, Amsterdam, Netherlands.; ^2^ Van ’t Hoff Institute for Molecular Sciences, University of Amsterdam, Science Park 904, 1090 GD, Amsterdam, Netherlands.; ^3^ Inorganic Chemistry and Catalysis, Debye Institute for Nanomaterials Science and Institute for Sustainable and Circular Chemistry, Utrecht University, Universiteitsweg 99, 3584 CG Utrecht, Netherlands.; ^4^ Antwerp X-ray Imaging and Spectroscopy laboratory, University of Antwerp, Groenenborgerlaan 171, 2020 Antwerp, Belgium.; ^5^ Paintings Laboratory, Royal Institute for Cultural Heritage (KIK-IRPA), Jubelpark 1, 1000 Brussels, Belgium.; ^6^ Photon Science at Deutsches Elektronen-Synchrotron DESY, Hamburg 22607, Germany.

## Abstract

*The Night Watch*, one of the most famous masterpieces by Rembrandt, is the subject of a large research and conservation project. For the conservation treatment, it is of great importance to understand its current condition. Correlated nano-tomography using x-ray fluorescence and ptychography revealed a—so far unknown—lead-containing “layer”, which likely acts as a protective impregnation layer applied on the canvas before the quartz-clay ground was applied. This layer might explain the presence of lead soap protrusions in areas where no other lead components are present. In addition to the three-dimensional elemental mapping, ptychography visualizes and quantifies components not detectable by hard x-ray fluorescence such as the organic fraction and quartz. The first-time use of this combination of synchrotron-based techniques on a historic paint micro-sample shows it to be an important tool to better interpret the results of noninvasive imaging techniques operating on the macroscale.

## Teaser

Rembrandt breaks tradition, employing lead-based impregnation for canvas preparation in *The Night Watch*.

## INTRODUCTION

*Militia Company of District II under the Command of Captain Frans Banninck Cocq*, the official title of *The Night Watch* (1642, Rijksmuseum, Amsterdam), is an important part of Rembrandt’s oeuvre. It is his largest (surviving) painting and is famous for the use of light and shadow to highlight different figures in the painting, thus enhancing the dramatic effect of the scene as a whole. Compared to the very static militia group portraits of his contemporaries, Rembrandt painted the group in action as they were starting to march, led by Captain Banninck Cocq and Lieutenant Willem van Ruytenburch, respectively, in the center left and center right (*1*).

Rembrandt’s masterpiece embodies four centuries of material history. During that period, the painting was subject to numerous chemical and mechanical alterations and other interventions (*1*). To determine the current condition of *The Night Watch*, it was necessary to understand the composition of the multilayered painting and the degradation processes taking place within the heterogeneous layer system; the latter comprises support, preparatory, paint, and varnish layers. With this goal in mind, *Operation Night Watch* was started in July 2019, the largest research and conservation project ever executed on this iconic work of art.

For *Operation Night Watch*, a wide variety of analytical and imaging techniques at the macro- and microscale are used to answer the following research questions: How did Rembrandt create *The Night Watch?* How did the materials change over time? What is the current condition of *The Night Watch?* And what is the best conservation treatment to ensure the long-term preservation of the painting? Within this broad investigation, the focus of this study lies specifically on the preparatory layers of *The Night Watch* on the microscale.

Historic paint systems are very heterogeneous, containing multiple organic and inorganic pigments in combination with an organic binder, such as linseed, poppy, or walnut oil. Microscale studies on paint samples are often performed on embedded paint cross sections (sample size around 50 to 500 μm) using microscopic imaging techniques, such as light microscopy (LM), scanning electron microscopy coupled with energy dispersive x-ray spectroscopy (SEM-EDX), attenuated total reflectance–Fourier transform infrared spectroscopy, μ-Raman spectroscopy, and synchrotron radiation (SR)–based 2D microscale x-ray diffraction (*2*, *3*). However, these two-dimensional (2D) imaging techniques only provide a partial picture of the shapes, sizes, and distribution of the pigment particles that are present below the visible surface plane of the cross section. This leads to a limited understanding of the morphology and composition of the paint and of its state of preservation. To improve our understanding of paint samples in 3D, we introduce the use of SR-based correlated x-ray fluorescence and x-ray ptychographic nano-tomography.

SR-based x-ray ptychography, to our knowledge, has so far not been applied to historical paint samples. In the literature, different microscale 3D imaging techniques have been reported for the investigation of paint samples (*4*). Gervais *et al.* (*5*) have shown the possibility of characterizing the porosity of the ground layer of a 19th-century painting using SR-based x-ray tomographic microscopy, using an energy of 17 keV. The pore size and interconnectivity between pores differed for the bottom, middle, and top part of the ground layer. Using full-field transmission x-ray microscopy (FF-TXM), Liu *et al.* (*6*) have shown that understanding the internal morphology of paint is very important to understanding transport processes within paints; these phenomena can lead to different types of degradation phenomena, while the morphology also affects the absorption of solvents used during conservation treatments. Previously, the authors of the current study have shown that FF-TXM at several energies can be used to identify degraded orpiment pigment based both on physical and chemical changes (*7*).

Next to the morphology, more specific chemical information from within a historical paint sample is of great interest. One approach uses x-ray powder diffraction tomography on historic paint samples of Van Gogh and Rembrandt (*8*–*10*). Although the number of studies using this technique is still limited, it already led to the discovery of a missing link in the case of red lead (Pb_3_O_4_) degradation (*9*) and helped to explain the formation of lead- and sulfur-containing surface crusts in paintings by Rembrandt (*10*).

Here, by applying x-ray ptychography on a historical paint sample, we explore what additional insights this technique can bring. Ptychographic x-ray–computed tomography (PXCT) is a coherent diffraction imaging (CDI) method. Far-field diffraction patterns are collected for a large number of overlapping positions by raster scanning a sample with a coherent x-ray beam at various rotation angles. An iterative ptychographic phase-retrieval algorithm is then used to provide the phase and amplitude of the wave field scattered by the object measured at each orientation. Tomographic reconstruction of the separate scans yields a 3D volume model of the electron density in the sample. An important characteristic of this CDI method is that the spatial resolution is not limited by the optical system that is used to define the primary beam (*11*–*13*). While working on microfossils, Maldanis *et al.* (*13*) have, for example, shown the potential of PXCT for 3D imaging at the nanoscale and exploited differences in electron density to segment the fossil filaments into several phases.

In our study, additional correlated SR-based x-ray fluorescence nano-tomography (SR-XRF nano-tomography) was collected, enabling us to produce a more complete 3D reconstruction of the sample by combining electron density and elemental information. The combination of these techniques has proven to be very useful in studies on biological organisms (*14*) and for the characterization of catalyst particles (*15*, *16*). In the case of the paint samples studied here, ptychography enables us to visualize the low-*Z* components of the paint that are not visible when using (hard) x-ray fluorescence imaging. The measurements were performed at beamline P06 at PETRA III (DESY) in Germany, a hard x-ray beamline (*16*–*21*).

In this study, we will focus on the preparatory layers that were applied to the canvas of *The Night Watch* before Rembrandt started to paint the scene that is visible today. It was the first time that he used a quartz-clay ground, a type of ground he would continue using until the end of his career, in alternation with other types of grounds (*22*). In the canvas paintings preceding *The Night Watch*, Rembrandt used a first ground layer of red earth [pigment rich in hematite (Fe_2_O_3_)], followed by a second ground layer containing lead white {pigment containing hydrocerussite [Pb_3_(CO_3_)_2_(OH)_2_] and cerussite (PbCO_3_)}, the most common white pigment in that period (*22*, *23*). The large size of *The Night Watch* may have motivated him to look for a cheaper, less heavy, and more flexible alternative for the ground layer. From the 1970s onward, the composition of Rembrandt’s grounds was thoroughly studied in the framework of the Rembrandt Research Project using (polarized) LM, SEM-EDX, and (small-angle) XRD (*22*, *23*). The aim of the present study is to gain more knowledge on the morphology and condition of the quartz-clay ground. In addition, we also discuss the added value of SR-based correlated x-ray fluorescence and x-ray ptychographic nano-tomography to the micro/nanoscale toolbox available for paint sample research. The results are supported by observations made using macroscale chemical imaging techniques that were used on *The Night Watch* as part of *Operation Night Watch*. These include macro–x-ray fluorescence (MA-XRF), macroscale x-ray powder diffraction (MA-XRD), and reflective imaging spectroscopy to visualize the distributions of respectively chemical elements, crystalline phases, and (colored) molecules, present as part of the original painting materials and their degradation products (*20*, *24*–*27*).

## RESULTS AND DISCUSSION

### Added value of SR-XRF nano-tomography in comparison to 2D SEM-EDX

**Fig. 1. F1:**
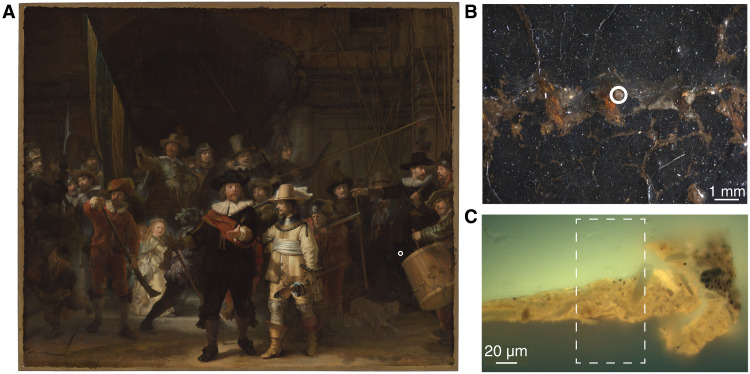
*The Night Watch* by Rembrandt van Rijn with the sample location and paint sample studied with correlated tomography. (**A**) *The Night Watch* by Rembrandt van Rijn (1642, Rijksmuseum Amsterdam, 378.4 cm by 453.0 cm) with an indication of the general sample area (white circle). (**B**) Microphotograph of the area corresponding to sample SK-C-5_003 (white circle) of the exposed quartz-clay ground (**C**) embedded paint fragment under a light microscope [ultraviolet (UV), 365 nm]. The white dashed rectangle shows the investigated area using correlated tomography.

The embedded paint fragment containing only the quartz-clay ground of *The Night Watch* shown in Fig. 1C was scanned using SR-based correlated x-ray ptychography and x-ray fluorescence nano-tomography. The paint sample was taken at a seam joining two strips of canvas, where the ground lies exposed due to paint loss. The original location of the fragment in the painting is shown in Fig. 1 (A and B). Figure 1C shows an optical micrograph. An area of 55 μm by 160 μm was scanned using correlated x-ray ptychography and XRF tomography, using 360 projections for the 3D reconstruction (Fig. 1C, dashed rectangle).

**Fig. 2.**
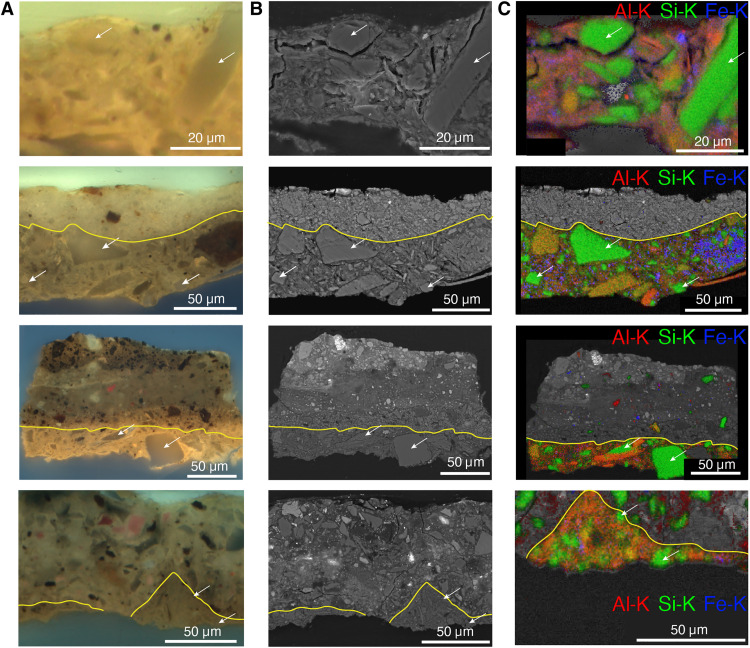
Comparison of the sample shown for correlated tomography (top row, sample, SK-C-5_003) with three other samples taken from *The Night Watch* containing the quartz-clay ground (second row: SK-C-5_008, third row: SK-C-5_062, fourth row: SK-C-5_030). [Fig F1]See fig. S1 for locations of origin of the samples. A yellow contour marks the interface between the ground and paint layers. (**A**) Light microscopy images under 365-nm UV light. (**B**) Backscattered electron images collected in the SEM. (**C**) Elemental distributions of aluminum, silicon, and iron as measured by SEM-EDX. Several quartz particles are indicated by white arrows.

To ensure that the studied sample was relatively representative of the ground layer throughout such a large painting, a comparison with other paint samples taken from *The Night Watch* containing the ground layer was made, as shown in Fig. 2. The samples were taken from different locations on the painting (see fig. S1). In both the ultraviolet (UV) and SEM image (Fig. 2, A and B), glassy quartz particles can be recognized (white arrows) within a matrix containing aluminosilicates, such as albite (NaAlSi_3_O_8_) and mica [e.g., KAl_2_(AlSi_3_O_10_)(F,OH)_2_], and goethite [FeO(OH)] (see Fig. 2C). All these minerals are often associated with clays. The four cross sections presented in Fig. 2 show that the ground layer has a similar composition in the different sampling locations, although the size of the quartz particles varies within and among the samples. Next to LM and SEM-EDX, MA-XRD and SR-μ-XRD were also used to characterize the composition of the ground layer. A table containing the different components found in the ground layer on *The Night Watch* can be found in the Supplementary Materials (table S1).

**Fig. 3.**
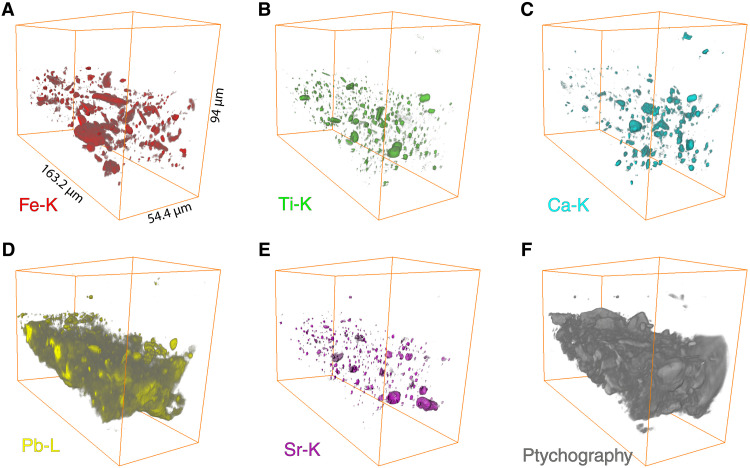
Three-dimensional rendering of several fitted elemental distributions of sample SK-C-5_003 as recorded by SR-XRF nano-tomography. (**A**) iron (Fe-K), (**B**) titanium (Ti-K), (**C**) calcium (Ca-K), (**D**) lead (Pb-L3), (**E**) strontium (Sr-K), and (**F**) the 3D rendering of the corresponding ptychography signal. See also movie S1.

Figure 3 shows the 3D reconstruction of the main elemental distributions of sample SK-C-5_003 as detected by SR-XRF: iron (Fe), titanium (Ti), calcium (Ca), lead (Pb), and strontium (Sr). Sorption of strontium to clay minerals is a well-documented phenomenon; this element is therefore used as a proxy to visualize the clay phase (*28*). In addition, in Fig. 3F, the 3D reconstruction of the collected ptychography signal is shown, corresponding to the low-*Z* components of the paint. See fig. S2 for the potassium (K), rubidium (Rb), manganese (Mn), nickel (Ni), copper (Cu), and zinc (Zn) distributions; their presence is not crucial for understanding the buildup of the sample.

**Fig. 4.**
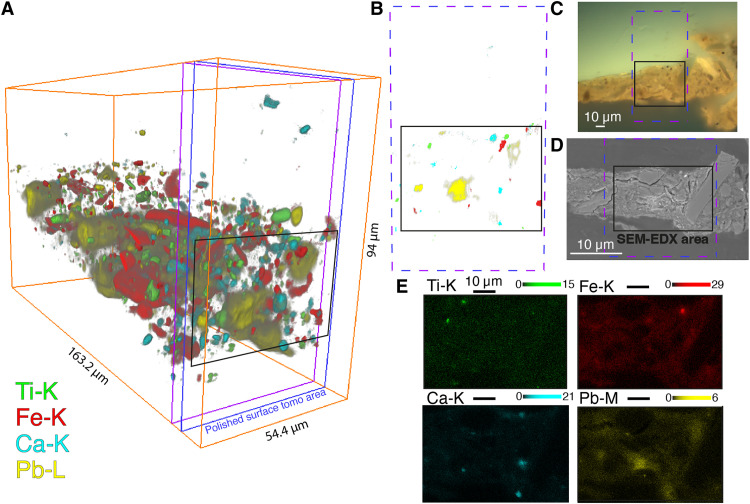
SR-XRF nano-tomography in comparison with SEM-EDX of sample SK-C-5_003. (**A**) Three-dimensional volume rendering of titanium (green), iron (red), calcium (cyan), and lead (yellow). The colors are scaled from gray to red, green, cyan or yellow. (**B**) Distribution of Ti, Fe, Ca, and Pb in the purple and blue slice indicated in (A), as measured by SR-μ-XRF. A threshold was used to show only the high intensity distribution. The black rectangle indicates the area scanned with SEM-EDX. (**C**) Light microscopy image of sample SK-C-5_003 under 365-nm UV light. (**D**) SEM image collected by circular backscattered (CBS) detector. (**E**) Elemental distributions of Ti, Fe, Ca, and Pb as measured by SEM-EDX.

In Fig. 4, the information obtained from SR-XRF nano-tomography is compared to that obtained by conventional 2D SEM-EDX mapping. SEM-EDX and previous research (*22*, *23*) on paint samples of the ground show that it contains many clay and mineral particles. Note that while aluminum, silicon, and magnesium are all difficult to detect with ambient air SR-XRF setups (and thus are not part of Fig. 3), they can straightforwardly be detected by SEM-EDX.

In Fig. 4A, four of the elemental 3D volumes are overplotted. The red color represents the net iron (Fe-K) XRF signal. Iron is present in particles of different sizes that can be divided into two groups: small particles with irregular shapes and two larger "plate"-shaped particles. The iron distribution in the SEM-EDX map in Fig. 4E shows that in this 2D view, only one small iron particle is detected. In the 3D rendering of the SR-XRF nano-tomography in Figs. 3A and 4A, we see that the sample contains many iron particles, much more than would be expected based on the single 2D SEM-EDX distribution. In the isolated view of iron in Fig. 3A, we clearly see the two large, flat “plate”-shaped particles containing a high concentration of iron. A great asset of the 3D rendering is that we can now visualize the complete particles and that their size is not underestimated because of missing information in one dimension.

If we consider the calcium distribution in Fig. 4 (B and E), then three relatively larger calcium-rich particles are present in the top, middle, and bottom parts of the sample. These particles confirm the correct alignment of the two measurement techniques. In Fig. 4A, the net titanium signal is represented in green. If we look at the titanium distribution in SEM-EDX in Fig. 4E, then we see no large titanium-containing particles present at the surface of the cross section. On the other hand, in the 3D rendering in Fig. 3B, we can distinguish different sets of particles: small spherical particles; small, elongated rod-like particles; and larger particles. The 3D results, e.g., of the iron and titanium-rich particles, indicate that SR-XRF nano-tomography can be used in future studies to identify individual crystallites, especially when combined with a complementary technique such as SR-μ-XRD. In this case, we assume that the large iron-containing "plate"-shaped particles are goethite [FeO(OH)], a mineral that was also identified at various locations in *The Night Watch* by means of MA-XRD. In one of the particles, there is a correlation between iron, titanium, and manganese; this is probably an ilmenite particle (see fig. S3).

The uppermost titanium-rich particle observed in SEM-EDX (Fig. 4E, top left) is not observed in the XRF scans in Fig. 4B. The titanium particle is probably present in a different tomographic slice than in the two slices shown in Fig. 4B. SEM-EDX probes a volume of the scanned object, of which the depth depends on the voltage used as well as the density and composition in the scanned area (*29*). Moreover, the sample is tilted at a slightly different angle in the tomographic view compared to the SEM-EDX scan. This makes it difficult to compare the two datasets in detail. Nevertheless, a comparison of the lead SEM-EDX map in Fig. 4E to the 3D lead distribution (Fig. 4A) clearly illustrates the improved sensitivity of SR-XRF for (high-*Z*) elements that are present in a low concentration: While the lead distribution in Fig. 4E shows a very low number of counts, the lead distribution is clearly visualized in the SR-XRF nano-tomography reconstruction.

**Fig. 5.**
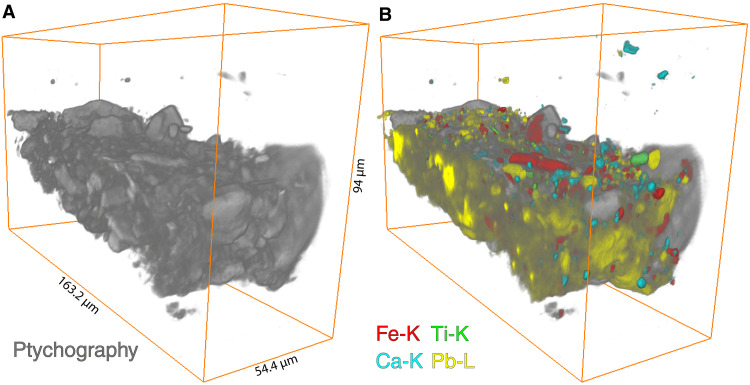
Three-dimensional renderings of the ptychographic reconstruction. (**A**) Three-dimensional rendering of the ptychographic reconstruction of sample SK-C-5_003. (**B**) Ptychographic reconstruction with the iron (red), titanium (green), calcium (cyan), and lead (yellow) distributions superimposed.

Simultaneously with the SR-XRF signal, ptychographic data were collected. As already mentioned, the measurement conditions do not allow the detection of elements such as carbon, aluminum, silicon, and magnesium with XRF due to the absorption of their characteristic radiation by the matrix as well as the ambient air between the sample and the XRF detector. Therefore, we are not able to visualize the organic fraction and other main components of the ground, such as quartz (SiO_2_) and aluminosilicates. However, as shown in Fig. 5A, x-ray ptychographic nano-tomography enables us to visualize these missing components in the paint sample. These include the organic fraction and the quartz particles. To guide the eye, in Fig. 5B, the 3D distributions of iron, titanium, calcium, and lead obtained by SR-XRF nano-tomography are shown together with the ptychographic rendering. The large iron-containing particles (red in Fig. 5B) can be easily recognized in Fig. 5A. The light gray particles visible in the front view of the sample in Fig. 5B are quartz particles, also indicated by the white arrows in Fig. 2 (top sample). The estimated 3D resolution of the XRF dataset is 591 nm and of the ptychography dataset 595 nm. The resolution estimates are based on the width of the line profiles obtained from sharp edges in slices of the reconstructed dataset and can be found in the Supplementary Materials (figs. S15 and S16).

The quartz particles cannot be easily recognized in the isolated, complete ptychographic reconstruction (Fig. 5A) because thresholding within the electron density histogram does not allow to separate the quartz particles from the other low-*Z* material. Therefore, volume masks were created on the basis of the distributions of Fe, Cu, Ca, Ti, Sr, or Zn in well-defined particles. The lowest part of the histogram was excluded as this is often correlated to noise or trace amounts within the organic fraction. These masks were used to remove voxels from the 3D ptychography rendering that contain chemical elements not related to the organic fraction. This resulted in a 3D volume corresponding to quartz, aluminosilicate-type minerals, or any other type of low-*Z* particles consisting of chemical elements below K (*Z* = 19).

**Fig. 6.**
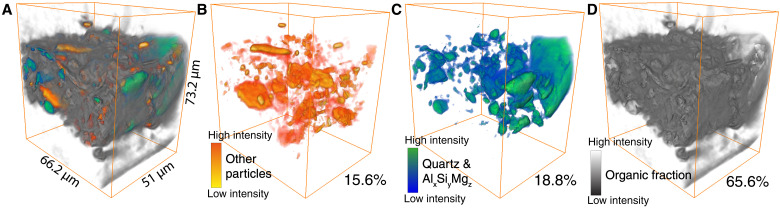
Three-dimensional rendering of ptychographic signal showing three volume fractions. (**A**) All volume fractions (total ptychographic signal) shown together. (**B**) Three-dimensional rendering of the particles observed in the ptychographic reconstruction that contain Fe, Cu, Ca, Ti, Sr, or Zn. (**C**) Three-dimensional rendering of quartz and aluminosilicates within the ptychographic reconstruction. (**D**) Three-dimensional rendering of the organic material and small particle fraction of the ptychographic reconstruction [i.e., the low electron density fraction that does not correlate with any of the elements within (B)].

In Figs. 3D and 6A, rendering of the ptychography reconstruction is shown, segmented in three volume fractions: high-*Z* (pigment) particles (ca. 15 vol %), quartz and aluminosilicates grains (ca. 20 vol %), and the organic fraction (ca. 65 vol %). The open pore space is not included in the volume calculation. It is notable to note that the volume percentage of quartz is much lower than the volume reported by Groen (*23*) (50 to 60 vol %). It is not exactly clear how Groen (*23*) calculated their percentages on the samples they studied, other than that it was based on SEM-EDX. We can calculate the particle size of the segmented quartz and alumina silicates. A large variety in particle size can be observed (see Fig. 6C and figs. S4 and S5). The large variation in the size of the quartz particles could be a cause of the substantial deviations in quartz percentages between this study and the work by Groen (*23*).

The quantitative analysis of the paint is important for heritage science for several reasons. First, it gives information about the creative process of the artist. Second, the percentages can also be used to make more realistic mock-up reconstructions to study, e.g., the original color and properties of the paint, as well as to perform aging tests. Last, information about the volume of the organic fraction is of importance for the prediction of the effect of cleaning methods used in conservation treatments (*30*).

### Unexpected presence of lead

**Fig. 7.**
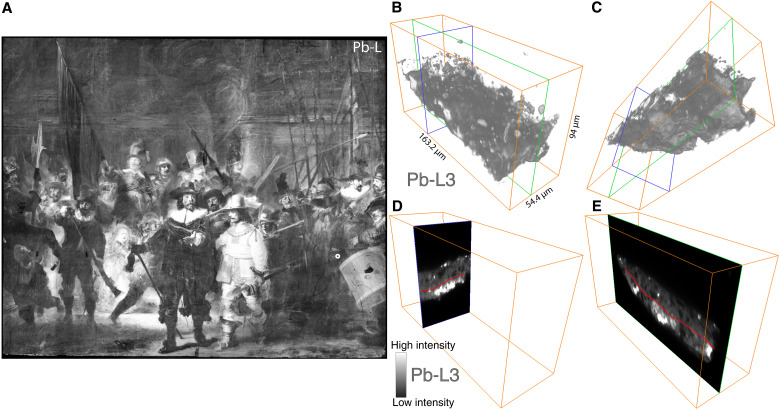
Three-dimensional rendering of the lead signal. (**A**) Lead Pb-L MA-XRF distribution map of *The Night Watch*. (**B**) Three-dimensional rendering of Pb-L intensity in the sample, looking down toward the top of the sample (i.e., closest to the surface of the painting) and (**C**) looking up toward the bottom of the sample (i.e., closest to the canvas). Two 2D orthoslices indicated (**D**) in blue and (**E**) green in the 3D volumes. The red line in (D) and (E) marks the lower, more homogeneous lead-containing "layer."

The stitched MA-XRF distribution map of lead (Pb-L; see Fig. 7A) of *The Night Watch* shows that generally, a lot of lead is present throughout the painting; while this is to be expected in the lighter parts of the composition where lead-containing pigments were used, e.g., the white collars, the faces, and the light yellow costume of Ruytenburch, it is also unexpectedly found in the dark background at the top of the painting. The lead in the background is mainly visible in the lead L-line distribution map and not in the lower energetic lead M-line distribution map. This shows that the lead that is present throughout the painting is in a deeper layer and not related to the visible composition. The lead that is present in the lighter parts of the composition is present in both L- and M-line distribution maps. The only layers that are considered to have been applied over the entire painting are the preparatory layers. It was not expected that the ground layer would contain substantial amounts of lead, as previous SEM-EDX measurements on cross sections of the ground layer of *The Night Watch* and of other Rembrandt paintings with a quartz-clay ground showed that only a limited amount of lead was present in this ground. Groen and van de Wetering (*22*) have mentioned lead white as a component of Rembrandt’s quartz-clay grounds; however, this identification appears to have been done only by SEM-EDX or via an unspecified analysis method. We therefore assume that in these analyses, the presence of lead was interpreted as the pigment lead white or a lead-based drier added to the oil binding medium; however, the results of our SR-based correlated x-ray fluorescence and ptychographic nano-tomography study have brought us to a new hypothesis to explain the presence of lead.

The first test scans at the synchrotron immediately showed that lead was present everywhere in the sample. Figure 7 (B and C) shows the 3D rendering of the lead signal. What is very notable is the difference in the distribution of lead throughout the sample, between the areas closer and further away from the paint surface. In the top part of the sample (i.e., the paint originally located at the surface, top in Fig. 7B), we observe that lead is present in individual particles, making the lead distribution comparable to that of other elements. In the bottom of the sample (i.e., closest to the painting’s canvas support), however, the distribution of lead is compact, much more homogeneous, and forms a “layer” (Fig. 7, B, D, and E); we assume that in this area, the lead is related to an organic fraction and not to a specific (inorganic) pigment.

**Fig. 8.**
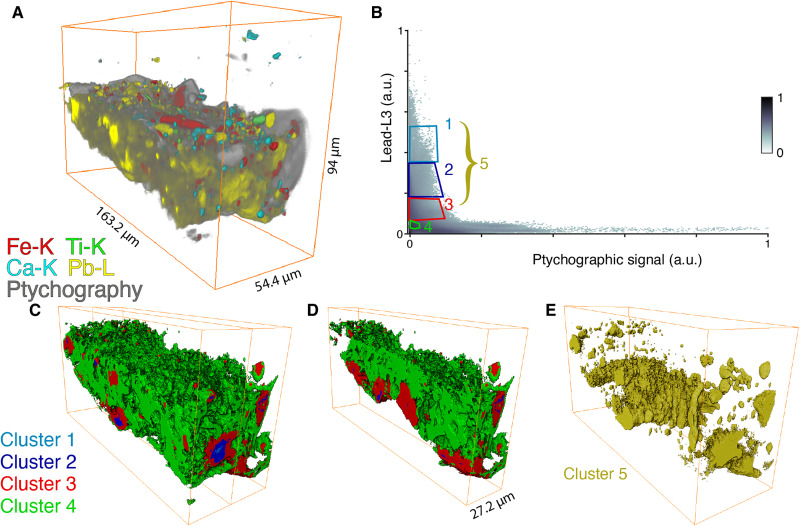
Three-dimensional rendering of the ptychographic reconstruction revealing the distribution of lead concentration. (**A**) Three-dimensional rendering of the ptychographic reconstruction. (**B**) Correlation plot between the ptychographic signal (*x* axis) and the lead (fitted Pb-L3) signal (*y* axis) (both in arbitrary units, a.u.). (**C**) Three-dimensional rendering of the first four clusters in which the green cluster correlates to the organic fraction containing a low concentration of lead and the red and blue clusters correlate to the lead migrating and accumulating in the ground layer, (**D**) virtual cross section of the 3D rendering along the middle, and (**E**) visualization of the combination of clusters 1 to 3 (supercluster 5).

Figure 8B shows the correlation between the ptychographic signal (*x* axis) and the Pb-L_3_ SR-μ-XRF intensity (*y* axis). An inverse correlation is visible, indicating that lead is mostly associated with low electron density regions. On the basis of this correlation plot, several clusters corresponding to different components of the sample can be defined. As shown in Fig. 8C, clusters 1, 2, and 3 correspond to areas with decreasing intensity of lead, whereas cluster 4 correlates to the organic fraction with a lower concentration of lead. We do not include the clusters that are high in lead concentration and electron density here. The red and blue clusters are mainly situated at the bottom part of the ground layer, while only some small accumulations of lead hotspots are visible in the upper area. By combining clusters 1 to 3 into a single supercluster (5), we are able to visualize the part of the ground layer that shows the largest lead accumulation in the organic fraction, as shown in Fig. 8E.

### Hypothesis

The presence of the more homogeneous lead-rich "layer" seen in Fig. 8E could be explained by the use of a lead compound as a drying additive to the oil used for paint preparation; this was a 17th-century practice used by Rembrandt and his contemporaries (*31*). In the sample under investigation, the lead intensity is mainly concentrated in the bottom part of the ground as can be seen in Figs. 7 (C to E) and 8E. If it was added to the oil used to prepare the ground or paint layers in this area of the painting, we would expect a fairly homogeneous distribution throughout the ground and paint layers. Since this is not the case, this suggests that a lead-containing "layer" is/was present underneath the quartz-clay ground.

**Fig. 9.**
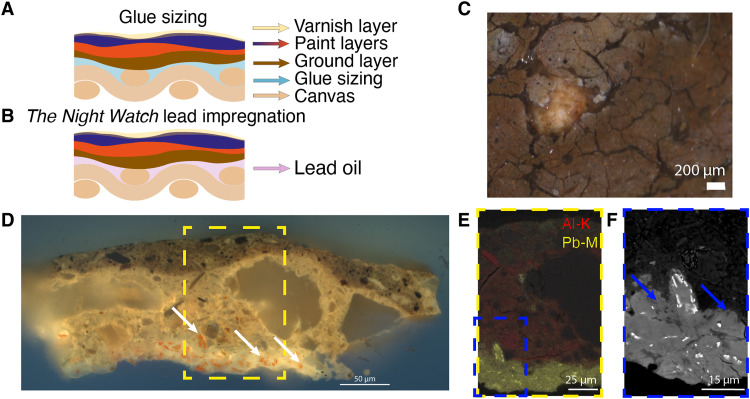
Various descriptions showing the lead oil impregnation "layer" between the canvas and the ground. (**A**) Schematic of a common build-up of a 17th-century oil painting using glue sizing. (**B**) Schematic build-up proposed for *The Night Watch* in which a lead oil was used to impregnate the canvas. (**C**) Stereomicroscopy image of a lead protrusion found on the surface of *The Night Watch*. (**D**) Light microscopy image of paint sample SK-C-5-093 under UV-A light (365 nm) from a paint fragment collected after the knife attack containing the lead-containing (luminescent) "layer" underneath the ground (the exact sample location in the painting is unknown). (**E**) SEM backscatter image overlaid with lead (Pb-M, yellow) and aluminum (Al-K, red) distribution collected with EDX (CBS/EDX, 20 kV, spot 5). (**F**) SEM backscatter image of the lead-containing "layer." The lead-containing hotspots have a high contrast (white). The blue arrows indicate two areas where dispersed lead has migrated into the ground layer. The quartz-clay ground can be recognized by the shapes of the clay crystallites with a lower contrast.

On the basis of this observation, we hereby propose another hypothesis about the origin of the lead-containing lower "layer." *The Night Watch* was originally commissioned for the *Kloveniersdoelen* (an important “musketeers’ shooting range” in 17th-century Amsterdam), where it hung in the “great hall” (*grote zaal* in Dutch) on the long exterior wall facing the windows. To protect the painting from humidity, we hypothesize that the canvas was impregnated with a lead-containing organic material, probably an oil before the ground layer was applied. In general, following the Netherlandish tradition of canvas preparation in the 17th century, one would expect a glue sizing of the canvas before the application of the ground layer (Fig. 9A). For this painting, Rembrandt appears to have deviated from this standard method. No indication of a glue-size layer has been found in any of the paint samples of *The Night Watch*, instead, the canvas seems to have been impregnated with a lead-containing oil. Figure 9B shows the proposed build-up of the painting. Although not generally used, the method of impregnating a canvas with lead-containing oil was described in an important contemporary source describing painting techniques written by Théodore de Mayerne (1573–1654/1655) (*32*). Here, the method of using a lead-rich oil (oil of litharge) instead of glue is advised after observing a case in which the paint and glue-sized canvas of a painting separated after hanging on a damp wall for several years. At least since the Middle Ages, lead, specifically red lead, in oil has been used to preserve stone, wood, and metal against humidity (*33*).

**Fig. 10.**
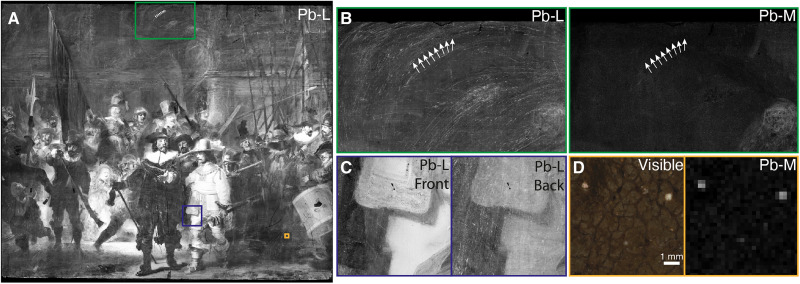
MA-XRF Pb maps demonstrating lead-rich strokes. (**A**) Lead Pb-L MA-XRF distribution map of *The Night Watch*, collected from the front. (**B**) Comparison of the front Pb-L and Pb-M MA-XRF maps (500-μm resolution) of an area with many lead-containing strokes. (**C**) Comparison with the Pb-L MA-XRF map collected from the front (250-μm resolution) and the back (500-μm resolution) of the painting, showing that the lead-rich strokes originate from a lower lying layer. (**D**) Two large lead soap protrusions observed in the visible photograph (5-μm resolution) and corresponding Pb-M MA-XRF map (250-μm resolution).

The MA-XRF Pb-L map shown in Fig. 10B shows that in the upper, dark background area of the painting, the lead-containing material appears to have been applied using large semicircular brushstrokes. The size, shape, and roughness of these strokes do not agree with recognizable painterly forms visible in *The Night Watch*. The pattern of the strokes and their occurrence in dark areas, as well as the absence of large lead-containing particles in the paint cross-sections from this area, demonstrates that the lead visible in the MA-XRF maps is not due to the use of a common lead-containing pigment such as lead white or lead-tin yellow. Note that the homogeneous distribution of lead in the nonfigurative parts of *The Night Watch* is much clearer in the MA-XRF map of lead Pb-L than in the Pb-M map (Fig. 10C). This shows that the homogeneous lead contribution originates from a lower lying layer in the painting, as the low energetic Pb-M radiation is strongly attenuated by the upper few micrometers of paint. The traces of the semicircular lead strokes also become clearer in the MA-XRF scan taken from the back (canvas side) of the painting with respect to the front, as shown in Fig. 10C, further strengthening our hypothesis that a lead-containing impregnation "layer" is present underneath the paint layers. We assume that this "layer" is present between the canvas and the ground layer, and not on the back of the canvas, due to features associated with the imprint of the original strainer bars onto which the canvas was originally stretched visible in the Pb-L distribution map (see fig. S6). This imprint results from a vigorous application of the lead-containing oil on the front, where the canvas was pushed against the strainer bars, causing an accumulation of material along the edges of the bars. In addition, during the wax-resin lining of the painting in 1975, the back of the original canvas was visible for some time; the treatment report mentions that in some areas where the canvas was worn, traces of a red material applied to the front of the canvas could be seen, supporting the hypothesis of a lead-containing impregnation "layer" between the canvas and the ground (*34*–*36*). During more recent stereomicroscopic observations, fibers of the original canvas support exposed in paint losses on the surface, have a reddish glow that is possibly associated with the oil impregnation "layer."

Following these observations on both the macro- and the nanoscale, the many paint fragments derived from the knife attack on *The Night Watch* in 1975 were critically re-examined regarding the presence of a red layer underneath the quartz-clay ground (*37*). Numerous paint fragments were identified showing clusters of red material underneath the ground layer. Figure 9D shows the LM image of one of these samples after embedding and preparation as a cross section under UV light (365 nm). The lead-containing impregnation "layer" is very clearly visible at the bottom of the sample, luminescent in the UV. The white arrows indicate red particles in this "layer". Figure 9E shows the SEM image of the yellow square in Fig. 9D, overlaid with the distribution of lead (yellow) and aluminum (red). The aluminum is an indicator of the (clay minerals in the) ground. The blue arrows in Fig. 9F indicate two locations where the dispersed lead has migrated into the ground layer, and the lead hotspots or red particles have high contrast (white). The red particles were identified by Raman spectroscopy as red lead (Pb_3_O_4_) (see fig. S7).

Lead ions have been shown to be very mobile in oil paint systems and can migrate across paint and varnish layers (*38*). This could explain why lead is found in the quartz-clay ground itself even though no lead-containing material is assumed to have been originally present there. These lead ions can quickly react with “free” fatty acids from the oil to form lead soaps. Because of their low solubility in oil paints (*39*), these lead soaps can crystallize within the paint and form aggregates that eventually can become so-called lead soap protrusions. A protrusion can disrupt the paint surface and become visible as a small white globule (*31*, *40*–*43*). This degradation phenomenon is well known in Old Master oil paintings (*44*–*48*).

In *The Night Watch*, many lead protrusions are present; these can even be observed visually as well as in the Pb-M MA-XRF maps (see Figs. 9C and 10D and fig. S8). Unexpectedly, the lead protrusions are found throughout the entire *The Night Watch*, including areas where so far, the presence of the lead (ions), required for their formation, could not be explained. The hypothesis of the presence of a lead-containing impregnation "layer" underneath the ground provides an explanation as to why lead (as observed by MA-XRF) and lead protrusions (visible by eye and stereomicroscopy) are present throughout the entire painting. Increased amounts of lead protrusions correspond to the semicircular lead-rich strokes. In addition, MA-XRD and SR-μ-XRD have shown the presence of other lead-containing degradation products in *The Night Watch* (e.g., lead sulfates, notably palmierite, K_2_Pb(SO_4_)_2_, which is extensively present in the painting) (*49*).

The migration of lead ions into the ground layer and the formation of lead soaps and lead soap protrusions may have been sped up and enhanced by a type of conservation treatment called “wax-resin lining” during which a melted wax-resin mixture was applied to the back of the painting to consolidate loose paint and to adhere a new canvas to support the original canvas. This conservation treatment was applied three times to *The Night Watch*: in 1851, 1945, and 1975 (*50*). The beeswax and (natural) resin mixture contained additional fatty acids (*51*, *52*); this mixture did not stay on the back of the canvas but was able to penetrate through the canvas and paint layers (*50*, *53*). We consider this has enhanced the formation of a homogeneous layer with elevated lead content in the bottom part of the ground (Fig. 7C). The additional free fatty acids that are introduced to the painting during the wax-resin lining treatment may also play a role in the formation of lead soaps. A preliminary study showed that when a wax-resin mixture (beeswax and colophony) was introduced into lead oxide, lead carboxylate species were formed (see fig. S9). The experiment was conducted at 40° and for a time of 70 hours, thus for a much longer time than during a typical wax-resin lining procedure, but it is important to note here that these materials can react.

The visualization of the lead layer in Fig. 8E supports the hypothesis that lead has migrated into the ground layer from below. The lower part of the lead distribution is where lead ionomers or lead soaps have formed. The lead-rich clusters in the upper part of the ground are presumably locations where lead ions or lead soaps have deposited and remineralized. This is confirmed by SR-μ-XRD measurements on sample SK-C-5_003 that reveal the presence of lead stearate (a lead soap) in the lower part of the sample (see fig. S10). Some of these crystalline lead carboxylates seem to have disappeared due to the very long exposure to x-rays during the tomography experiment. On the basis of the lead distribution at the start and end of the experiment, no migration of lead species took place (see fig. S11).

This study shows that SR-based correlated x-ray fluorescence and ptychographic nano-tomography is a promising and useful new tool to visualize a sample of the quartz-clay ground layer used by Rembrandt in *The Night Watch*. This advanced 3D (chemical) imaging method provides much more detail and additional knowledge about the paint sample morphology and condition in comparison to conventional 2D studies of paint cross sections. Some characteristic mineral particles or crystallite morphologies such as "plate"-like shapes are very difficult to observe using conventional 2D microscopic analysis methods, while they are readily visible in the 3D volume data. This technique is potentially useful to study, in great detail, the differences in reactivity exhibited by pigment particles based on their shapes and sizes. The ptychography results enabled us to visualize the organic fraction as well as the quartz particles and other low-*Z* components of the paint, thus allowing for a more complete 3D reconstruction of the paint sample in comparison to only SR-XRF nano-tomography. The technique also allowed us to estimate the volume and particle sizes of the quartz and aluminosilicate particles, as well as that of the organic fraction and the pigments and other mineral grains.

In this study, the presence of a thus far unknown lead-containing impregnation "layer" in Rembrandt’s iconic masterpiece *The Night Watch* was revealed, located below the ground layer. The information gained by SR-based correlated x-ray fluorescence and ptychographic nano-tomography in combination with that of MA-XRF at the macroscale supports the hypothesis that the canvas of *The Night Watch* was impregnated with lead-rich oil instead of preparing it with a more traditional glue sizing. The presence of this lead-containing impregnation "layer" helps to further elucidate the current condition of *The Night Watch*, especially to explain the unusual presence of lead protrusions in areas of the painting where no other lead-containing compounds were used in the stratigraphy of the paint. At this time, there are a number of limitations for correlated SR-based correlated x-ray fluorescence and ptychographic nano-tomography to become a more commonly used analytical method for paint cross-section research. These are mainly related to the long data collection time, the large amount of data collected, and the elaborated data processing procedure, being both computer- and labor-intensive. Some of these limitations and their possible solutions or alternatives are discussed in the Supplementary Materials. Given that some of these limitations are likely to be overcome in the near future, both by the evolution of synchrotron source brilliance and computer processing capacity, we foresee that SR-based correlated x-ray fluorescence and ptychographic nano-tomography will become a valuable tool for future cultural heritage studies.

## MATERIALS AND METHODS

### Sample preparation

A small paint fragment was taken from “The Night Watch” with a scalpel and with the help of a stereomicroscope. The sample was embedded in Technovit 2000 LC mounting resin (Heraeus Kulzer GmbH, Germany). This is a one-component methacrylate that cures under visible blue light (Technotray CU, Heraeus Kulzer GmbH, Germany). The embedded sample was then wetly polished using a Struers LaboSystem polishing machine with flushing water and silica abrasive paper (grades 320 up to 2400) using a MOPAS polishing holder to hold the sample. Subsequently, the cross section was further dry polished using Micro-Mesh sheets up to grade 12,000 (Micro-Surface Finishing Products Inc., Wilton, Iowa, USA) (*54*).

### Light microscopy

LM of the embedded paint cross sections was carried out on a Zeiss Axio Imager.A2m microscope equipped with a Zeiss AxioCam MRc5 digital camera. The cross sections were analyzed at magnifications of up to 500×, in bright field, and UV (UV-A, 365-nm LED light source Zeiss Colibri 2). The luminescent images were collected by using a filter cube composed of a 365-nm excitation filter (EX G 365), a beam splitter at 395 nm (BS FT 395), and an emission long-pass filter at 420 nm (EM LP 420).

### Scanning electron microscopy–energy dispersive x-ray analysis

The samples were analyzed using a FEI NovaNano SEM 450 scanning electron microscope. For the backscattered electron images, an acceleration voltage of 15 or 20 kV was used, at high vacuum conditions (circular backscattered detector, samples SK-C-5_008 and _062a) or low vacuum conditions (GAD detector, samples SK-C-5_003 and _030). Elemental maps were collected after applying a coating of 3 nm of tungsten using a Leica EM ACE600 Sputter Coater, with a voltage of 20 keV by the Thermo Fisher Scientific UltraDry EDS Detector using the Thermo Fisher Scientific Pathfinder x-ray Microanalysis Software for data processing.

### Correlated x-ray fluorescence and ptychographic nano-tomography

The correlated x-ray fluorescence and ptychographic tomography experiment of sample SK-C-5_003 was performed at beamline P06 at PETRA III, DESY, Hamburg (Germany). The energy of the monochromatic x-ray beam was 17 keV and the beam size was 200 nm (*V*) by 300 nm (*H*). KB focusing optics were used to focus the beam. The sample was placed on a rotating stage in the center of rotation. An area of 55 μm by 160 μm of the sample was raster scanned. The scans were collected with a step size of 200 nm and a dwell time of 2 ms was used. From 0° to 180° and from 180.5° to 360.5°, every degree a scan was collected, bringing the total amount of scans to 362. Acquisition time per scan was around 8 min, bringing the total acquisition time to around 48 hours. XRF data of every scan was collected by two SII Vortex EM Si-drift detectors oriented perpendicular to the primary beam on either side of the sample. Ptychographic diffraction patterns were recorded with an Eiger X 4 M hybrid pixel detector (Dectris Ltd.) placed at a distance of about 8 m from the sample.

A fitting model of the XRF data was made using PyMCA (*55*). The fitting model was used to fit the entire XRF dataset. The sum XRF spectrum of all measurements of one of the rotations is shown in fig. S12 together with the used fit model. The fitted images of every projection of the two detectors were combined by adding the images together. During the reconstruction, two angles were excluded because they showed too many artifacts. Some projections contained outlying pixels that showed a very high intensity; these were filtered out by replacing the value of the pixel with the average of the neighboring pixels using MATLAB. For a few elements, there was a cluster of high-value pixels; in this case, the value of the outlying pixels was replaced by a threshold value based on the histogram of the 2D image, again using MATLAB.

In every scanning point, a far-field diffraction pattern was collected. The ptychographic data were reconstructed using an in-house method using the ePIE algorithm combined with a phase retrieval framework, using 1000 iterations (*11*, *56*).

The achieved pixel size of the ptychography reconstruction in 2D was 45.3 nm. The 2D ptychography maps were cropped to exclude the edges of the reconstructed images, as the reconstruction in these areas was not satisfactory (see figs. S13 and S14 for the histogram of the image). Some projections showed too many artifacts in the whole image and were excluded for the reconstruction, bringing the total of used projections to 324. These 2D images were then loaded into TXM Wizard for 3D reconstruction.

### 3D reconstruction

TXM Wizard software (*57*) was used to align a common feature in all projections and an iterative Algebraic Reconstruction Technique (i-ART) (i = 20) algorithm was used for the 3D reconstruction of both the XRF and ptychographic projections images. For the XRF reconstruction, we used two common features to improve the alignment.

Avizo software was used for the visualization and handling of the 3D reconstructions. The segmentation and calculations of the components was also done using Avizo.

An estimation of the achieved spatial resolution in 3D was calculated by measuring line profiles along sharp edges in orthoslices retrieved from the 3D volume. More information on this method can be found in the Supplementary Materials. The achieved average 3D spatial resolution was 591 nm for the XRF dataset and 595 nm for the ptychographic data.

## Supplementary Materials

### This PDF file includes:

Supplementary Text Figs. S1 to S16 Table S1 Legend for movie S1 References
