# Supplementary material for: Correlated x-ray fluorescence and ptychographic nano-tomography on Rembrandt’s The Night Watch reveals unknown lead “layer”
Source: Sci Adv. 2023 Dec 15;9(50):eadj9394. doi: 10.1126/sciadv.adj9394 (PMC10848709; doi:10.1126/sciadv.adj9394)
Supplement: Supplementary file 1 — Supplementary Text Figs. S1 to S16 Table S1 Legend for movie S1 References [file sciadv.adj9394_sm.pdf]

Science Advances
AAAS

Supplementary Materials for

Correlated x-ray fluorescence and ptychographic nano-tomography on Rembrandt's *The Night Watch* reveals unknown lead "layer"

Fréderique T.H. Broers *et al.*

Corresponding author: Fréderique T.H. Broers, f.t.h.broers@uva.nl; Florian Meirer, f.meirer@uu.nl

Sci. Adv. **9**, eadj9394 (2023)
DOI: 10.1126/sciadv.adj9394

The PDF file includes:

Supplementary Text
Figs. S1 to S16
Table S1
Legend for movie S1
References

Other Supplementary Material for this manuscript includes the following:

Movie S1

Supplementary Text and Supplementary Figures

Additional information samples of the ground of *The Night Watch*

Figure S1 shows the painting *The Night Watch* by Rembrandt van Rijn. The white circles indicate the locations where paint samples were taken during *Operation Night Watch*. These samples are used to make a comparison of the ground layer in these samples.

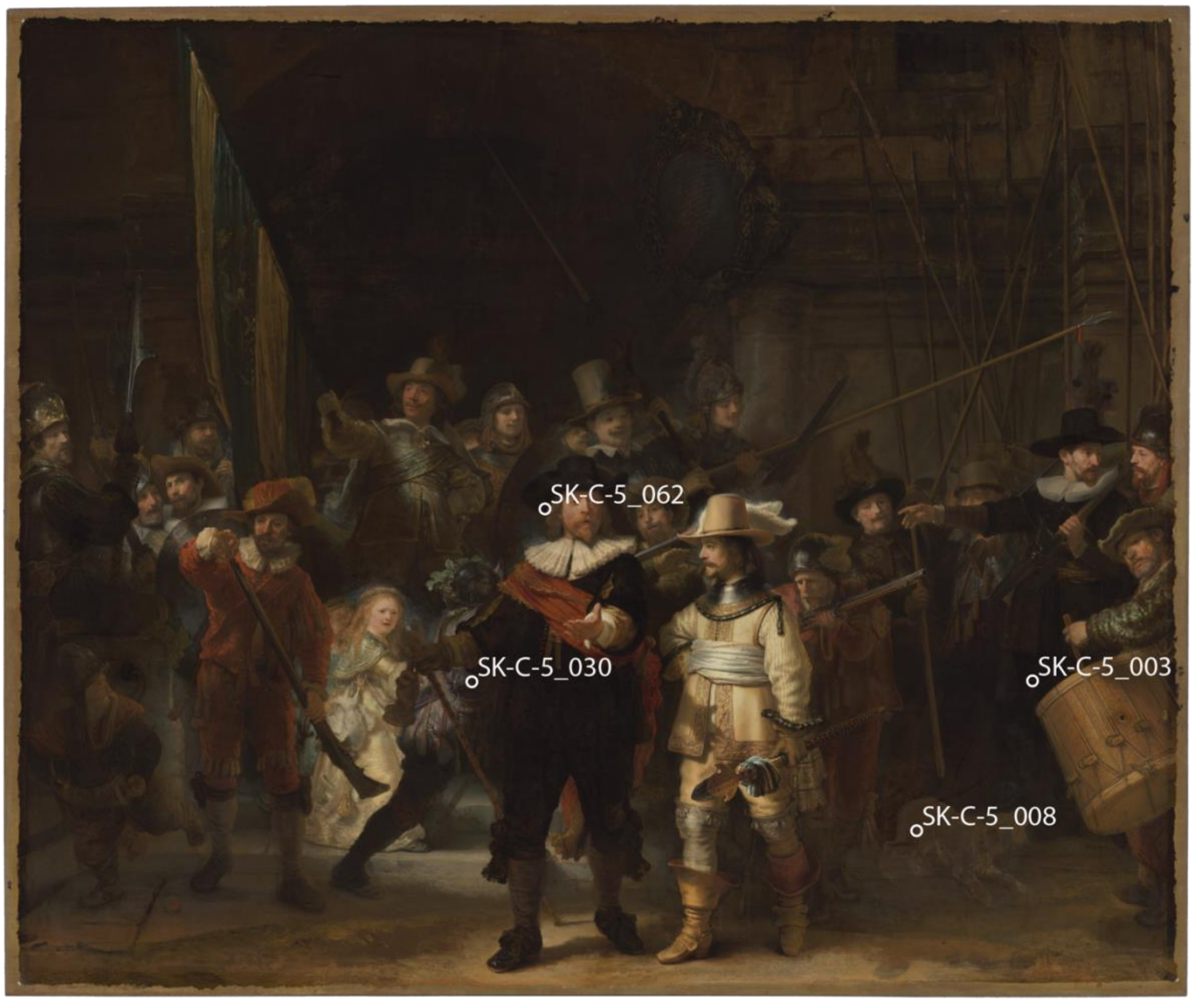

Figure S1.
***The Night Watch* by Rembrandt van Rijn from 1642, oil on canvas, 379.5 cm by 453.5 cm, Rijksmuseum, Amsterdam (SK-C-5) with locations where samples SK-C-5_003, SK-C-5_008, SK-C-5_030, and SK-C-5_062 were taken.**

Inorganic composition of Rembrandt's quartz-clay ground

The material used for the ground of *The Night Watch* consists mainly of quartz and clay minerals. In Table S1 the identified components of the quartz-clay ground are listed, together with the method of identification. Iron is part of the ground due to its presence in different clays. Iron is also a component of the mineral goethite (α-FeO(OH)). MA-XRD on the painting confirmed the presence of goethite in areas where the ground is exposed. Furthermore, SR-μ-XRD studies performed on paint samples at beamline P06 showed the presence of celadonite ($K(Mg,Fe^{2+})(Fe^{3+},Al)[Si_4O_{10}](OH)_2$, mica group mineral), a component of the pigment green earth.
Fig. S2f shows the 3D rendering of the potassium (K) signal collected by tomographic XRF. Potassium is heterogeneously distributed throughout the main part of the sample volume. Potassium is a very common element in minerals and can substitute e.g. sodium atoms in albite (feldspar), a mineral detected in Rembrandts' ground by MA- and SR-μ-XRD studies. The newly formed mineral is called K-feldspar or microcline.

Table S1.
Composition of the quartz-clay ground, identified as part as *Operation Night Watch* and previous analytical studies on *The Night Watch*

Components	Chemical formula	Identification by	Remarks
Quartz	SiO_2	LM(*23*, *58*), SEM-EDX(*23*, *58*), MA-XRD (*23*, *58*), SR-μ-XRD(*23*, *58*) 09/11/2023 13:49:00	Alpha-quartz
Albite	$NaAlSi_3O_8$	MA-XRD(*23*, *58*), SR-μ-XRD(*23*, *58*)	Part of feldspar group
Goethite	α-FeO(OH)	MA-XRD(*23*, *58*), *RIS*(*23*, *58*)	Reflectance Imaging Spectroscopy (RIS)
Illite	$(K,H_3O)(Al,Mg,Fe)_2(Si,Al)_4O_{10}[(OH)_2\cdot(H_2O)]$	SEM-EDX(*22*), SR-μ-XRD(*23*, *58*)	
Gypsum	$CaSO_4\cdot 2H_2O$	SR-μ-XRD(*23*, *58*)	
Calcite	$CaCO_3$	MA-XRD (*23*, *58*), SR-μ-XRD(*23*, *58*)	
Muscovite (?)	$KAl_2O_{10}(OH)_2$	SEM-EDX(*22*), petrography(*22*)	Mica category
Phlogopite (?)	$KMg_3AlSi_3O_{10}(F,OH)_2$	SEM-EDX(*58*)	Mica category – very small amounts of F (SEM-EDX)
Kaolinite (?)	$Al_2(OH)_4Si_2O$	XRD(*23*)	Could be part of goethite

Charcoal	C	LM(*58*), SEM-EDX(*58*)	

(?) Exact species is difficult to determine

Fig. S2c shows the 3D rendering of calcium in the sample. Calcium is mainly present as gypsum and calcite; this mineral was identified with both MA-XRD as well as SR-μ-XRD. Calcite is often used in paints as a filler next to being employed as a white pigment. In many soils and clays, calcium carbonates are also present. Either the calcite and gypsum were added to the clays used for the ground layer or were already present in the clay used for the ground.

MA-XRF results showed the presence of titanium in areas where the ground was exposed. With MA-XRD, no crystalline components containing titanium were found at the surface of the paint; however, SR-μ-XRD studies performed on cross sections of *The Night Watch* demonstrate that rutile or anatase were present in the ground. Whereas quartz and albite ($NaAlSi_3O_8$) were always identified in the ground layer samples, this was not the case for anatase or rutile (TiO_2). The 3D rendering of titanium in Fig. 2b shows that titanium is definitely present in the ground. We can distinguish different sets of particles: small spherical particles, small elongated rod-like particles and larger particles. As the XRF set up used in this study is not intended/optimized to detect light elements, we can unfortunately not correlate the titanium distribution to that of oxygen. Looking into the correlation of iron with titanium, we only found very few particles showing a correlation (See Fig. S3). In these particles, also some manganese is present. The correlation between these elements suggests that this might be ilmenite, a titanium-iron oxide mineral ($FeTiO_3$), containing considerable amounts of Mn. Combined SEM-EDX and XRD research conducted in previous research identified ilmenite in many quartz-clay grounds used by Rembrandt, however not in the samples from *The Night Watch*. Manganese is detected in low intensity in many particles that also contain one or more of the main elements. A small number of particles of zinc and copper are detected.

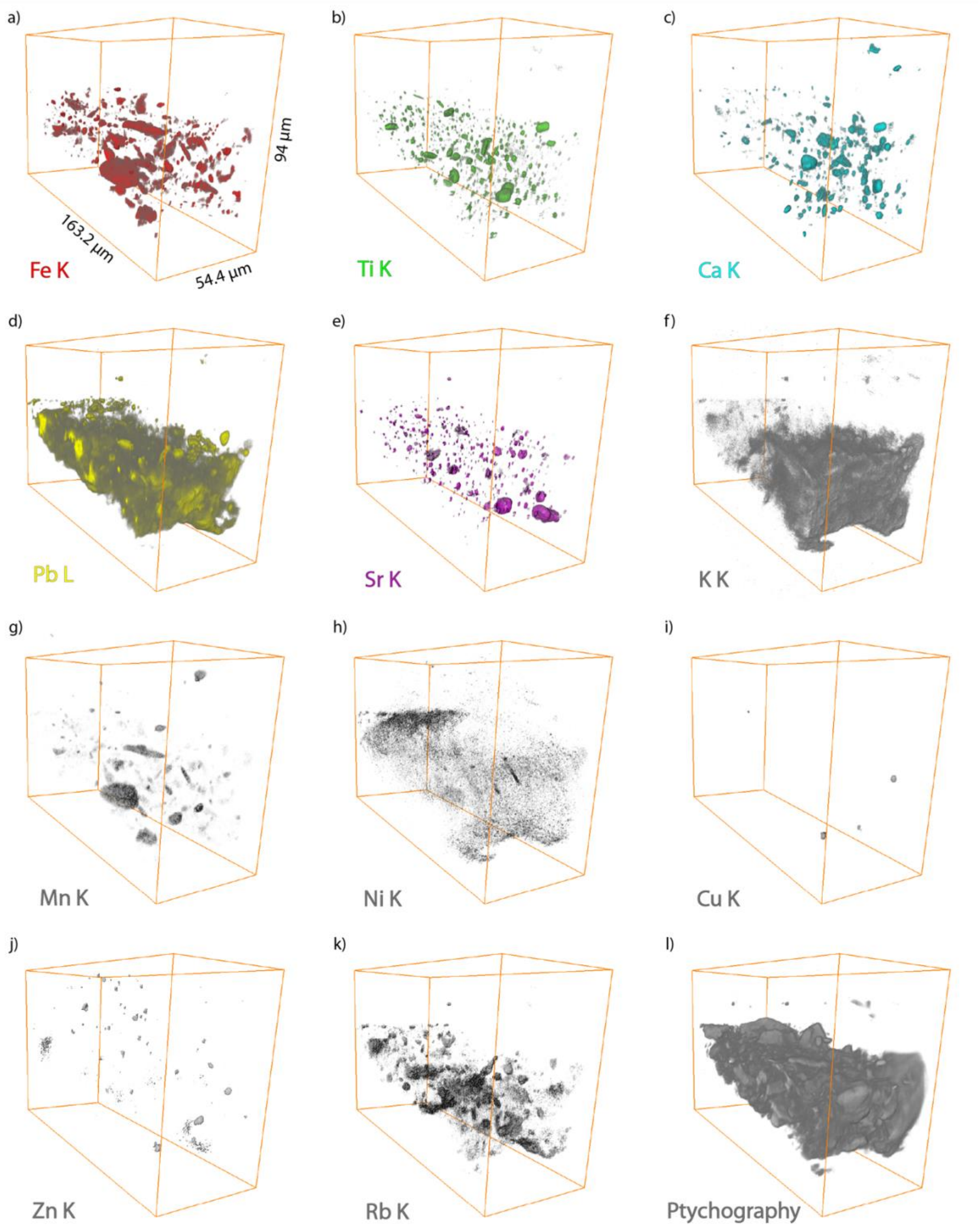

Figure S2.

3D rendering of the different fitted elemental distributions of the sample of the ground of *The Night Watch* as recorded by SR-XRF nano-tomography. The elemental distribution of a) iron (Fe-K), b) titanium (Ti-K), c) calcium (Ca-K), d) lead (Pb-L3), e) strontium (Sr-K), f) potassium (K-K), g) manganese (Mn-K), h) nickel (Ni-K), i) copper (Cu), j) zinc (Zn-K), k) rubidium (Rb-K) and l) the 3D rendering of the ptychograph signal.

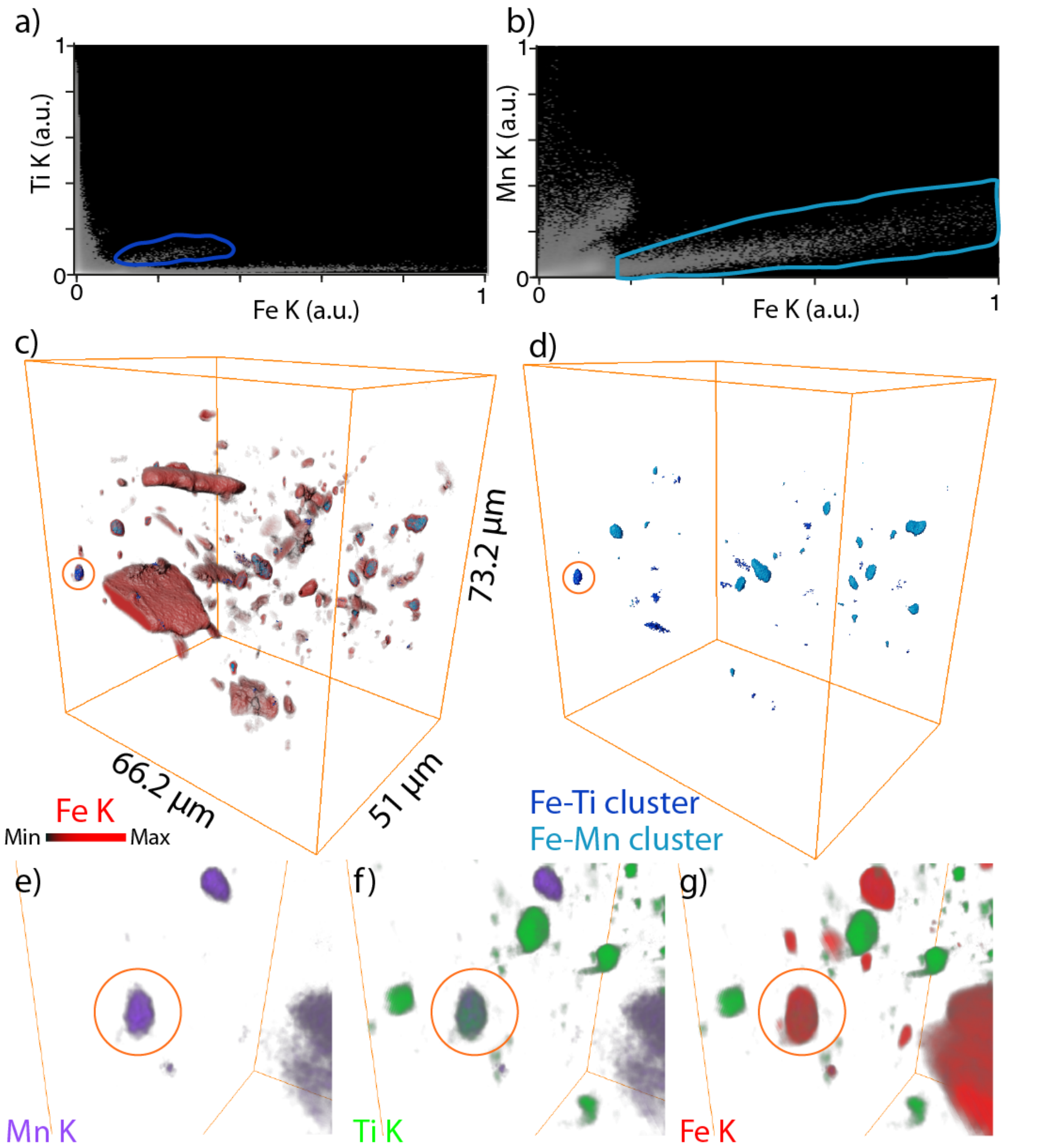

Figure S3.

Correlation study between iron, titanium and manganese. a) Correlation plot with Fe-K on the X axis and Ti-K on the Y axis. The cluster of voxels with a correlation between Fe and Ti is encircled in dark blue. b). Correlation plot with Fe-K on the X axis and Mn on the Y axis b) 3D rendering of the Fe K signal in red. The orange circle indicates the ilmenite particle. c) 3D rendering of the four clusters indicated in a. e) Zoom in on the ilmenite particle in the orange circle, showing the distribution of Mn in purple, f) Ti in green and g) Fe in red.

Particle size distribution of quartz and aluminosilicate particles

As described in the main text, the ptychography results were used to segment the quartz and aluminosilicate particles. Fig. S4 shows the separate particles in randomized colors. Fig. S5 shows the particles size distribution in voxels.

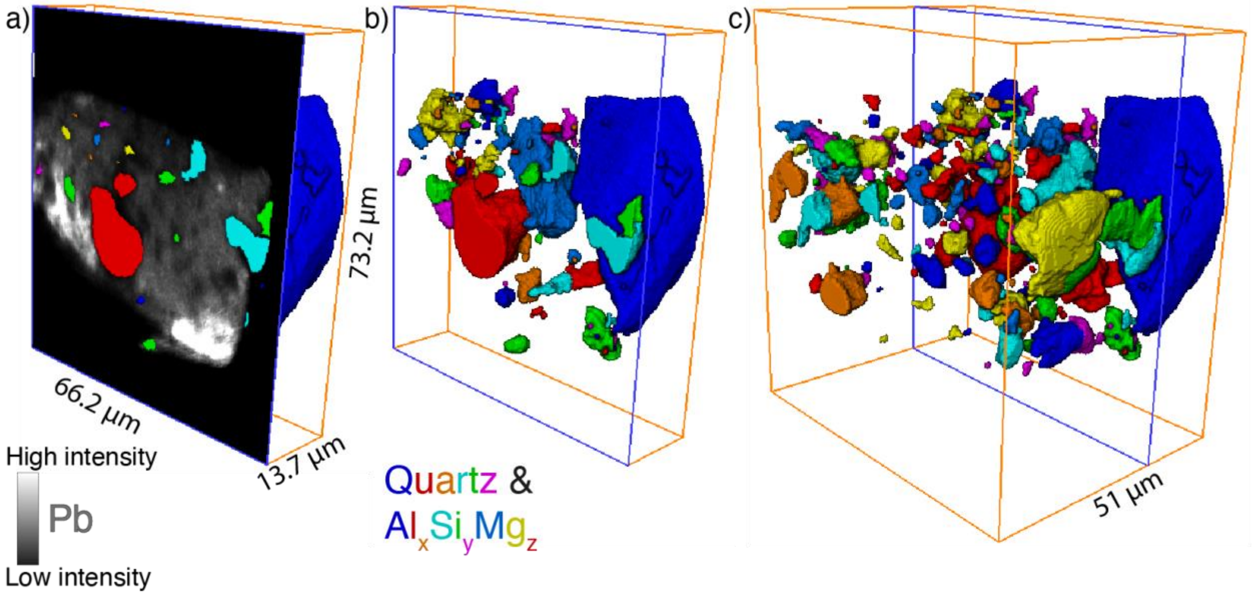

Figure S4.
Segmentation of the quartz and aluminosilicate particles in 3D. a) Lead distribution in grey and segmented quartz and aluminosilicate particles in randomized colors b,c) 3D rendering of quartz and aluminosilicate particles.

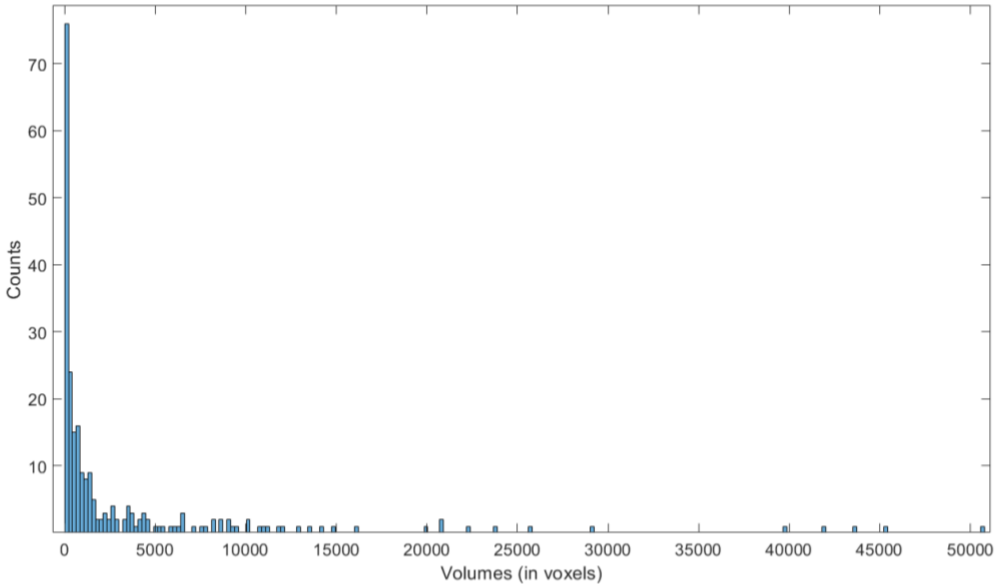

Figure S5.
Distribution of particle size of quartz and $Al_xSi_yMg_z$, excluding three outliers with a volume above 75000 voxels. Each voxel is 200 x 200 x 200 nm.

Additional results supporting the presence of a lead containing layer

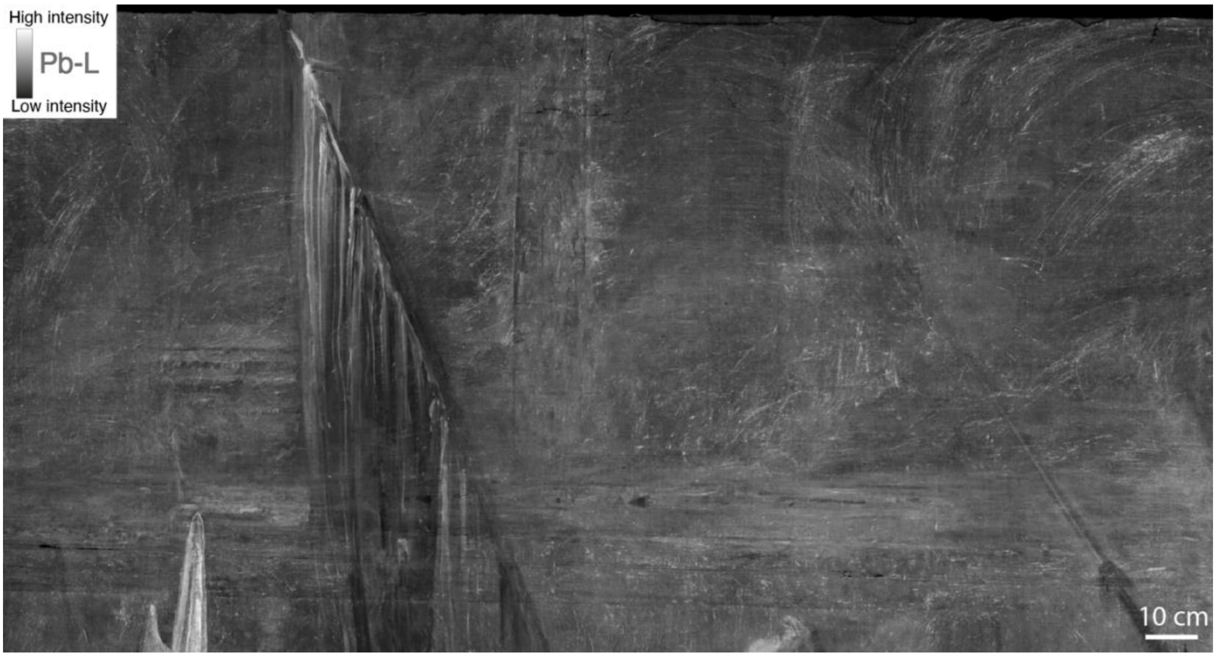

Figure S6.
MA-XRF lead L distribution map collected during *Operation Night Watch.* In this detail from the upper left, imprints of the original stretcher bars (vertical bar in the middle of the image) are visible due to the pressure exerted by the bars against the canvas when the lead-containing oil was applied.

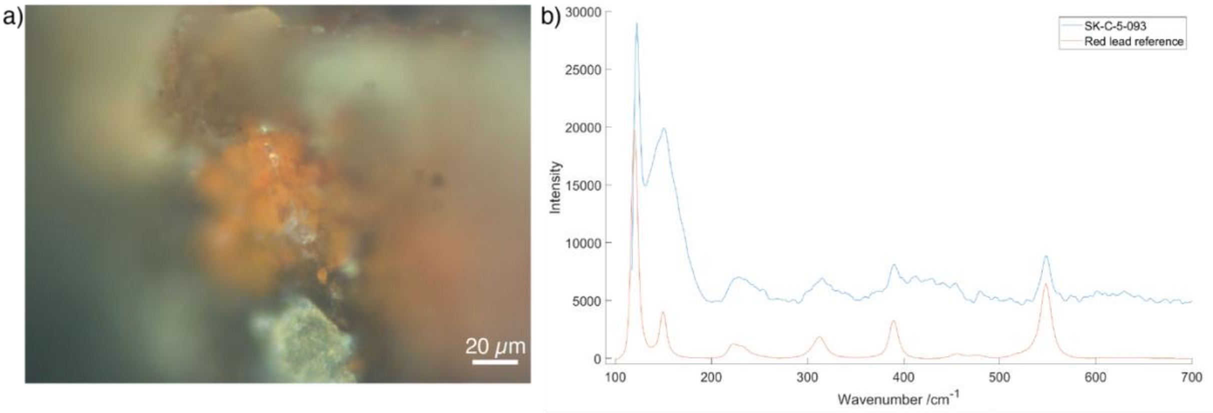

Figure S7.
Presence of red lead confirmed with Raman spectroscopy. a) Light microscopy image of bottom of sample (SK-C-5-093) that was collected after the knife attack in 1975 on *The Night Watch*(*37*) b) The Raman spectrum collected on the red particles is shown in blue and a reference spectrum of red lead is shown in red.

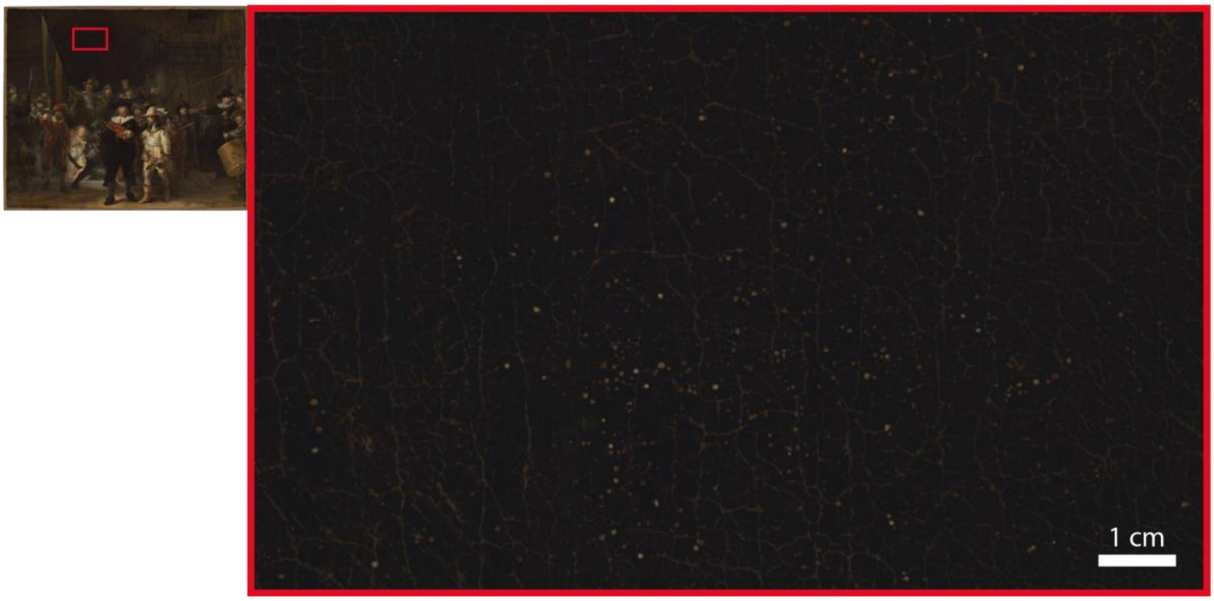

Figure S8.
Lead protrusions (white spots) observed on the surface of the painting under normal light 5 µm photography.

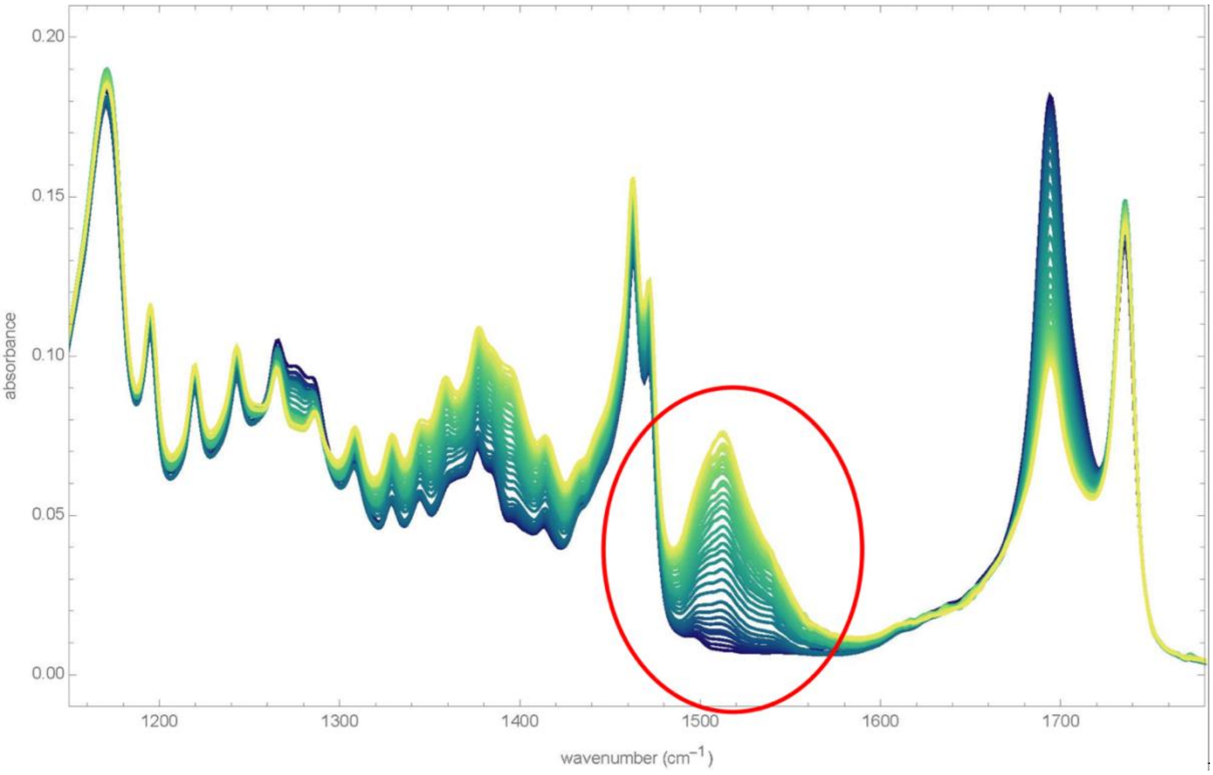

Figure S9.
Formation of lead carboxylates after introduction of a wax resin mixture to lead oxide. The lead carboxylate band is located between 1490–1670 cm^{-1}. The time series is shown from dark blue to yellow and spans a period of 70 hours at a temperature of 40 degrees Celsius.

Beam damage

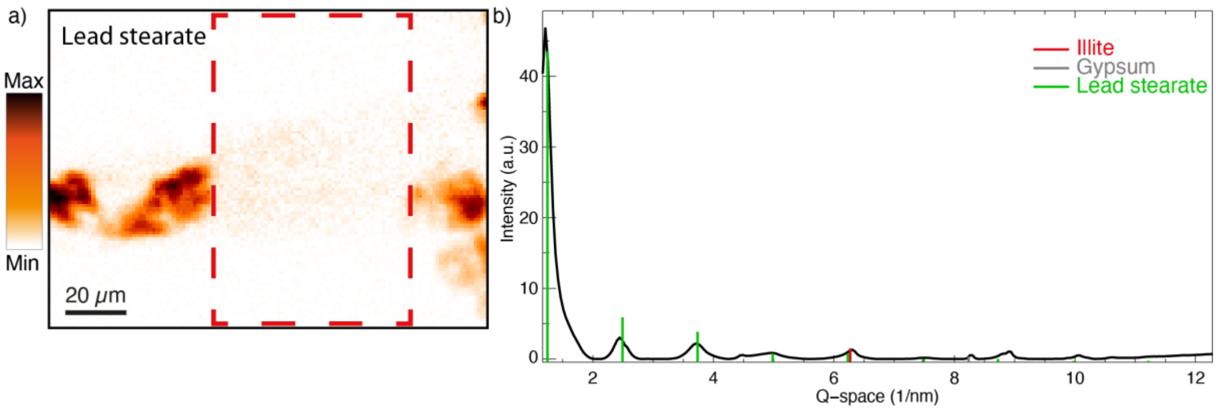

Figure S10.
Confirmation of the presence of lead stearate in the sample of the ground. a) Distribution of lead stearate in the sample as measured by SR-μ-XRD. The red dashed rectangle shows the scanned area during the tomography experiment. The very high X-ray dose that was applied to this area has caused the disappearance of crystalline lead stearate. b) Average XRD pattern of the lead stearate area shown in a) with superimposed the PDF of illite (red), gypsum (grey) and lead stearate (green).

Fig. S10a shows the distribution of lead stearate in sample SK-C-5_003 as collected by SR-μ-XRD. This data was measured at beamline ID13 during beamtime allocated to BAG proposal HG-172. A scan of 95 by 130 μm was collected with a dwell time of 10 milliseconds and a step size of 1 μm in both directions with an energy of 12.95 keV. It should be noted that the XRD data were collected after the tomographic measurements. The XRD map reveals the absence of lead stearate within the area covered by the tomography measurements. We attribute the disappearance of lead stearate to the high dose of radiation the material was exposed to during the X-ray tomography scan (more than 48 hours) at the synchrotron. Lead soaps are prone to decompose after (extensive) exposure to highly intense synchrotron X-ray beams. This was previously observed by the authors in a paint sample of *Girl with a Pearl Earring* by Johannes Vermeer (1665, Mauritshuis, The Hague)(*2*). Distortions or movements of the sample due to instability or beam-induced changes were checked by comparing the projection at 0 to the projection at 180 degrees and the projection at 180.5 degrees with the one at 360.5 degrees (see Fig. S11). While slight tilting of the sample was noted, especially during the first 180 scans, no significant (elemental) changes of the sample were observed.

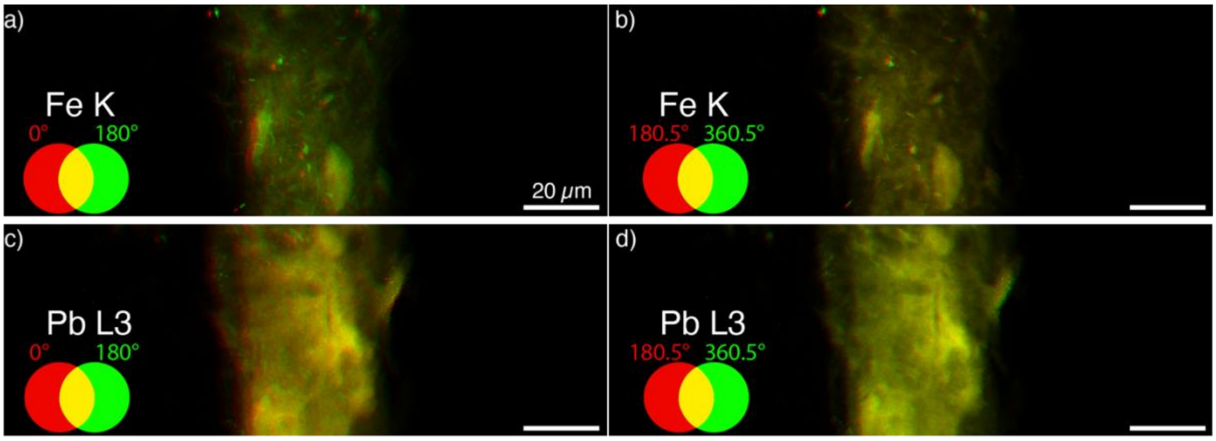

Figure S11.

Study into beam-induced changes. 2D fitted elemental distribution of the sample at 0° (red) and 180° (green) for a) iron, c) lead andat 180.5° (red) and 360.5° (green) for b) iron and d) lead. A small misalignment can be seen after 180-degree rotation in both the Fe-K and Pb-L3 distribution.

The small differences can mainly be related to a small error in the alignment of the images, detector noise and the use of two detectors at the opposite sides of the sample. The changes are more prominent for the comparison of the sample after the first 181 scans (see Fig. S11 a and c). This might also be explained because after 159 scans, the measurements had to be stopped for about 12 hours due to technical maintenance of the synchrotron electron beam. During this time, the hutch was opened and the sample might have shifted slightly on the stage. This probably accounts for most changes observed between 0° and 180°. Fig. S11b and d show that less changes are observed when comparing the scan at 180.5° and 360.5°.

Limitations of correlative ptychography and XRF tomography

The described technique comes with some limitations for it to become a more commonly used analytical technique. At this time, the measurements are time consuming and results in a large amount of raw data. The ptychographic reconstructions require a lot of computing power. Therefore, measurements can only be done on a limited number of samples and relatively small sample volumes. Beam-induced alterations can also occur during the tomography experiment due to long exposure times of the sample to the beam. The results of such an analysis for the sample in this study can be found in the supporting information (Fig. S11 section 'Beam Damage'). No notable elemental changes due to the beam were observed, but we did observe some loss of crystallinity.

For answering certain research questions, transmission X-ray microscopy might be an alternative for visualizing a complete sample in 3D in comparison to ptychography. TXM requires less post-collection data processing. Another option could be to repeat the XRF study of a sample at both a hard and soft X-ray beam line to obtain information from low Z elements. A downside would be that the sample must be much smaller to avoid over-absorbance of the beam in the soft X-ray regime. To speed up the measuring time, fewer angles could be measured at a loss of tomographic resolution. If a quantification of the electron density is desired, we would advise to scan the sample on a pin instead of inside an embedding medium. To identify components with similar elemental composition, correlated tomographic XRD could be collected. We expect that ongoing developments in synchrotron brilliance, automatized data processing and increasing computing power will overcome these limitations in the (near) future.

Fit model XRF

Figure S12 shows the sum XRF spectrum of all pixels collected in one angle (0°) during the XRF tomography together with the fit model created in PyMCA.(*55*)

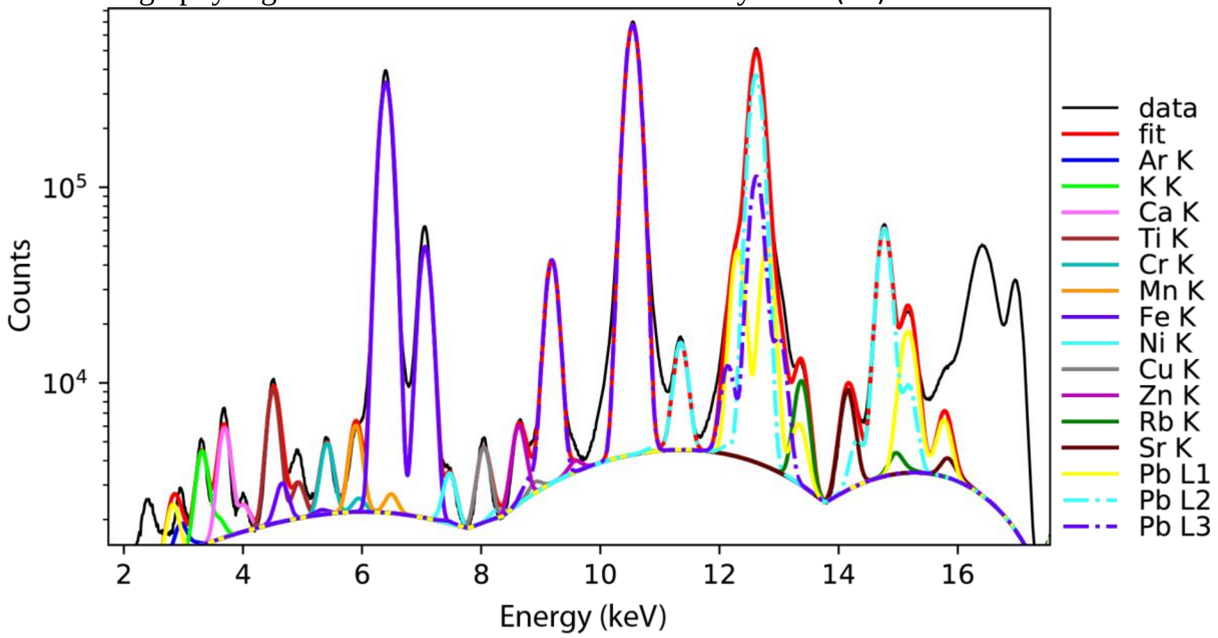

Figure S12.
Sum XRF spectrum of all pixels collected at angle 0 (black), the fit model (red) and the fit lines of the individual elements (for colors see legend).

Segmentation of ptychography data

In Figure S13, the result of one of the ptychography projections after the iterative reconstruction is shown. To the left and right of the sample the embedding medium can be seen, while the center of the image shows the strong intensity originating from the sample. In conservation/heritage science it is common practice to embed the small and precious paint fragments in a small resin block. In this study, Technovit 2000LC was used. This methyl acrylate cures with the use of blue light. Due to the presence of this embedding medium, we do not have air in the measured area which can be used as background. Fig. S14 shows the histogram of the ∂ (the refractive index decrement, which represents the change in phase velocity, see Wittwer *et al.* (*11*) for more detailed information) of the 3D dataset shown in Fig. 5a. There are no distinguishable Gaussians in the histogram that would relate to different components based on a difference in electron density between materials. Different efforts to use thresholding to differentiate the lower part of the electron density to separate the contribution from the embedding medium, the oil used for the paint, and conservation treatment materials such as the wax resin lining were unsuccessful. This was probably due too similar electron densities of these organic materials. Additionally, the embedding material absorbs part of the X-rays before they reach the samples and measuring an embedded sample is therefore not the best procedure for ptychography, especially if you want to look at very subtle differences in electron density as is the case for the organic fractions.

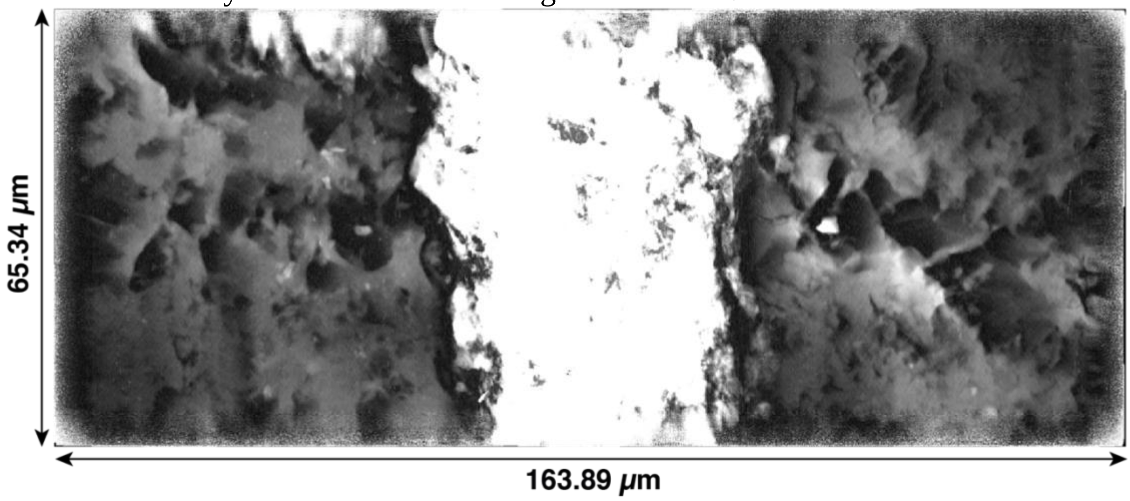

Figure S13.

Reconstructed 2D X-ray ptychography projection which resulted after 1000 iterations using the ePIE algorithm (*11*). At the edges of the image, information is lacking for a correct reconstruction. Left and right of the sample signal from the embedding medium is observed. The contrast of the image is enhanced for clarity.

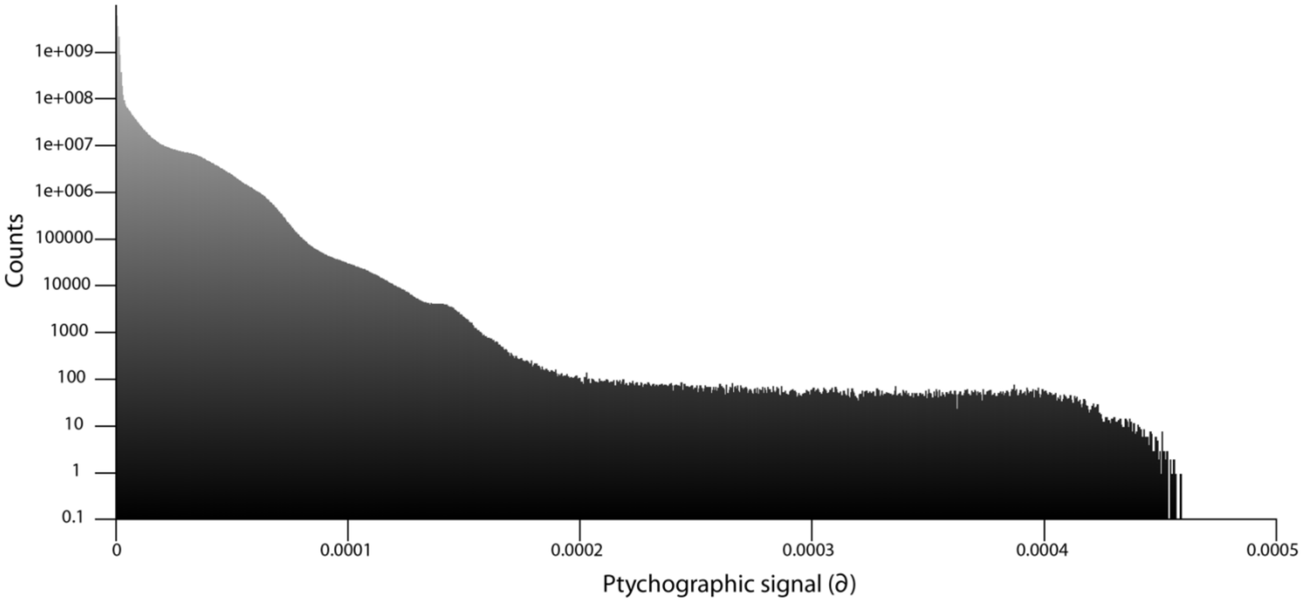

Figure S14.
Histogram of the ptychography data in Fig. 5a. The x-axis shows the ∂ (refractive index decrement). The y-axis shows the number of counts (in logarithmic scale).

Estimation of spatial resolution of the 3D reconstructions

To estimate the spatial resolution of the reconstructed 3D volumes, line profiles of sharp edges on ortho slices of both the 3D XRF data set and ptychography data set were measured. A Gaussian profile was fitted over the change in intensity of the grey values along the line. This was done by using an in-house developed MATLAB script. More details on the method can be found in Bossers *et al.* (*15*) and Wise *et al.* (*19*)

Figure S15 shows the spatial resolution estimation of the XRF results, calculated on sharp edges within ortho slices of the copper (Fig. S15a) and strontium (Fig. S15b) data sets. These elements were selected due to their high contrast in particles and low contrast in the background, unlike an element such as lead of which low concentrations are present in many of the voxels in the 3D data set. The achieved average 3D spatial resolution for the XRF data set was 591 nm.

Figure S16 shows the spatial resolution estimation of the ptychographic reconstruction. Two line profiles were measured in the central ortho slice from the 3D reconstructed ptychography dataset. The achieved average 3D spatial resolution of the ptychography dataset was 595 nm.

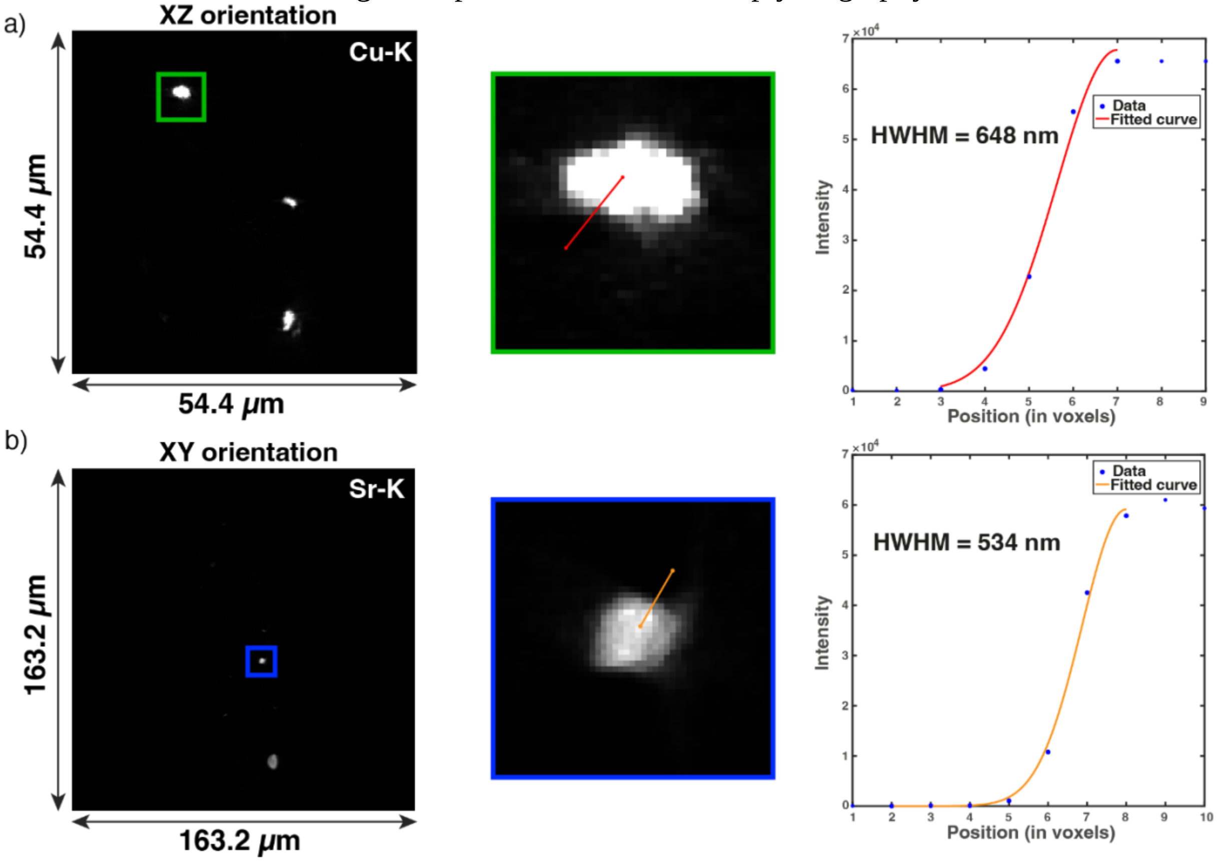

Figure S15.

Study of the 3D spatial resolution of the XRF dataset. a) Reconstructed ortho slice of the Cu-K data set in XZ orientation (voxel size 200 nm). The green box shows a zoom-in of the area where a line profile (red line) was measured. The graph on the right shows the data points on this line and the fitted Gaussian profile in red, the calculated half width half maximum (HWHM) is taken as an estimation of the achieved resolution of the 3D dataset. b) Reconstructed ortho slice of the Sr-K data set in XY orientation (voxel size 200 nm). The blue box shows a zoom-in of the area where a line profile (red line) was measured. The graph on the right shows the data points on this line and the fitted Gaussian profile in red, the calculated half width half maximum (HWHM) is taken as an estimation of the achieved resolution of the 3D dataset.

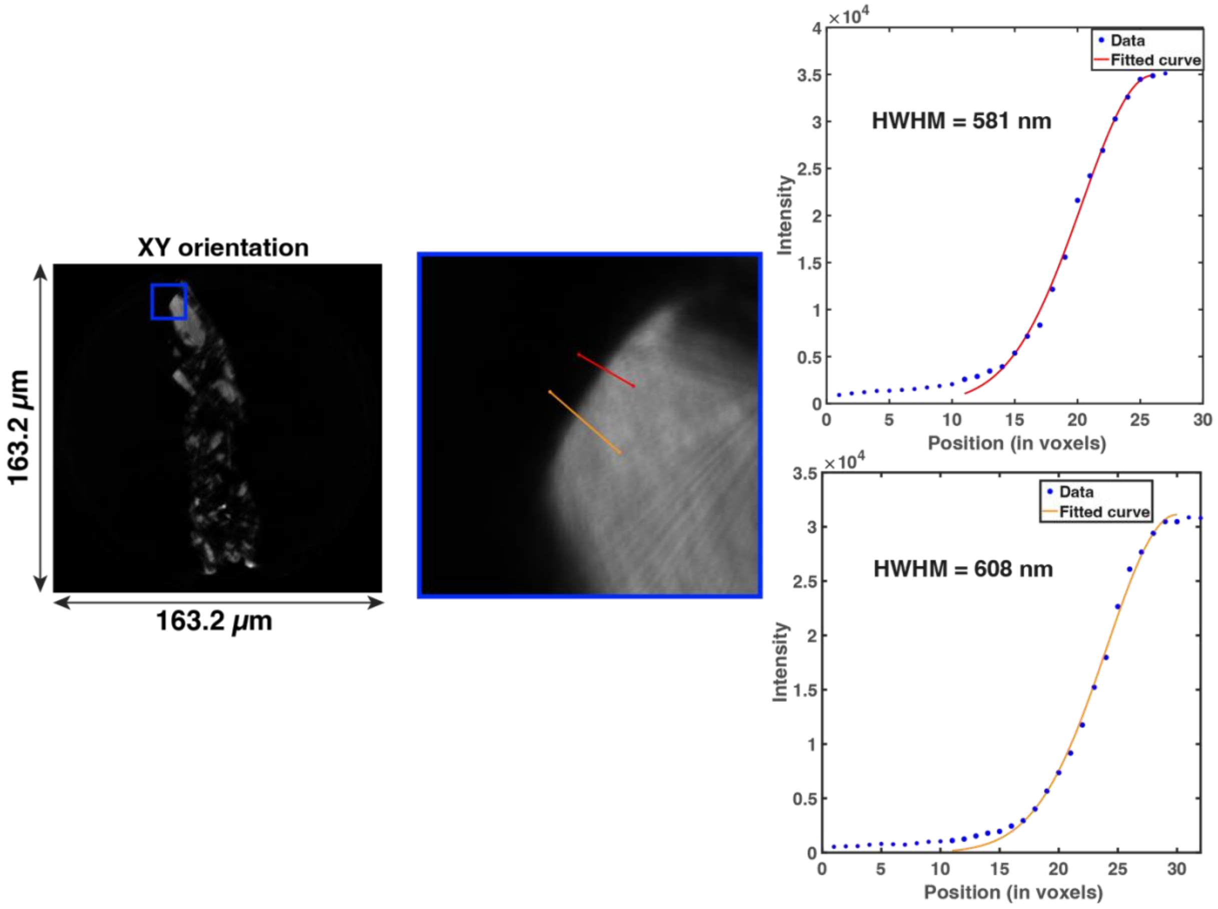

Figure S16.

Study of the 3D spatial resolution of the ptychographic dataset. On the left a reconstructed ortho slice of the ptychography data set in XY orientation is shown (voxel size 45.3 nm). The blue box shows a zoom-in of the area where two line profiles (red and orange line) were measured. The graphs on the right show the data points on these lines and the fitted Gaussian profile in red and orange, the calculated half width half maximum (HWHM) is taken as an estimation of the achieved resolution of the 3D dataset.

Movie S1.

3D rendering of Pb (L3) distribution in yellow, Ca (K) distribution in cyan, Fe (K) distribution in red, Ti (K) distribution in green and ptychographic signal in grey.

REFERENCES AND NOTES

1. J. Bruyn, The ‘Night watch’, or officers and men of the company of Captain Frans Banning Cocq and Lieutenant Willem van Ruytenburgh, in *A Corpus of Rembrandt Paintings III*, M. Nijhoff Ed. (Springer, 1989), pp. 430–485.

2. A. van Loon, A. Vandivere, J. K. Delaney, K. A. Dooley, S. De Meyer, F. Vanmeert, V. Gonzalez, K. Janssens, E. Leonhardt, R. Haswell, S. de Groot, P. D’Imporzano, G. R. Davies, Beauty is skin deep: The skin tones of vermeer’s girl with a pearl earring. *Herit. Sci.* **7**, 102 (2019).

3. L. Monico, S. Prati, G. Sciutto, E. Catelli, A. Romani, D. Q. Balbas, Z. Li, S. De Meyer, G. Nuyts, K. Janssens, M. Cotte, J. Garrevoet, G. Falkenberg, V. I. Tardillo Suarez, R. Tucoulou, R. Mazzeo, Development of a multi-method analytical approach based on the combination of synchrotron radiation X-ray micro-analytical techniques and vibrational micro-spectroscopy methods to unveil the causes and mechanism of darkening of “fake-gilded” decorations in a Cimabue painting. *J. Anal. At. Spectrom* **37**, 114, 129 (2022).

4. M. Cotte, A. Genty-Vincent, K. Janssens, J. Susini, Applications of synchrotron X-ray nano-probes in the field of cultural heritage. *Comptes Rendus Physique* **19**, 575–588 (2018).

5. C. Gervais, J. J. Boon, F. Marone, E. S. B. Ferreira, Characterization of porosity in a 19th century painting ground by synchrotron radiation X-ray tomography. *Appl. Phys. Mater. Sci. Process.* **111**, 31–38 (2013).

6. X. Liu, V. Di Tullio, Y. C. Lin, V. De Andrade, C. Zhao, C. H. Lin, M. Wagner, N. Zumbulyadis, C. Dybowski, S. A. Centeno, Y.-C. Karen, C. Wiegart, Nano- to microscale three-dimensional morphology relevant to transport properties in reactive porous composite paint films. *Sci. Rep.* **10**, 18320 (2020).

7. F. T. H. Broers, K. Janssens, J. Nelson Weker, S. M. Webb, A. Mehta, F. Meirer, K. Keune, Two pathways for the degradation of orpiment pigment (As_2S_3) found in paintings. *J. Am. Chem. Soc.* **145**, 8847–8859 (2023)

8. W. De Nolf, F. Vanmeert, K. Janssens, *XRDUA* : Crystalline phase distribution maps by two-dimensional scanning and tomographic (micro) X-ray powder diffraction. *J. Appl. Cryst.* **47**, 1107–1117 (2014).

9. F. Vanmeert, G. Van der Snickt, K. Janssens, Plumbonacrite identified by X-ray powder diffraction tomography as a missing link during degradation of red lead in a van gogh painting. *Angew. Chem. Int. Ed.* **54**, 3607–3610 (2015).

10. S. W. T. Price, A. Van Loon, K. Keune, A. D. Parsons, C. Murray, A. M. Beale, J. F. W. Mosselmans, Unravelling the spatial dependency of the complex solid-state chemistry of Pb in a paint micro-sample from Rembrandt's Homer using XRD-CT. *Chem. Commun.* **55**, 1931–1934 (2019).

11. F. Wittwer, J. Hagemann, D. Brückner, S. Flenner, C. G. Schroer, Phase retrieval framework for direct reconstruction of the projected refractive index applied to ptychography and holography. *Optica* **9**, 295 (2022).

12. F. Pfeiffer, X-ray ptychography. *Nat. Photonics.* **12**, 9–17 (2018).

13. L. Maldanis, K. Hickman-Lewis, M. Verezhak, P. Gueriau, M. Guizar-Sicairos, P. Jaqueto, R. I. F. Trindade, A. L. Rossi, F. Berenguer, F. Westall, L. Bertrand, D. Galante, Nanoscale 3D quantitative imaging of 1.88 Ga Gunflint microfossils reveals novel insights into taphonomic and biogenic characters. *Sci. Rep.* **10**, 8163(2020).

14. J. Deng, Y. H. Lo, M. Gallagher-Jones, S. Chen, A. Pryor, Q. Jin, Y. P. Hong, Y. S. G. Nashed, S. Vogt, J. Miao, C. Jacobsen, Correlative 3D x-ray fluorescence and ptychographic tomography of frozen-hydrated green algae. *Sci. Adv.* **4**, eaau4548(2018).

15. K. W. Bossers, R. Valadian, S. Zanoni, R. Smeets, N. Friederichs, J. Garrevoet, F. Meirer, B. M. Weckhuysen, Correlated X-ray ptychography and fluorescence nano-tomography on the fragmentation behavior of an individual catalyst particle during the early stages of olefin polymerization. *J. Am. Chem. Soc.* **142**, 3691–3695 (2020).

16. K. W. Bossers, R. Valadian, J. Garrevoet, S. van Malderen, R. Chan, N. Friederichs, J. Severn, A. Wilbers, S. Zanoni, M. K. Jongkind, B. M. Weckhuysen, F. Meirer, Heterogeneity in the fragmentation of ziegler catalyst particles during ethylene polymerization quantified by X-ray nanotomography. *JACS Au.* **1**, 852–864 (2021).

17. S. Kalirai, U. Boesenberg, G. Falkenberg, F. Meirer, B. M. Weckhuysen, X-ray fluorescence tomography of aged fluid-catalytic-cracking catalyst particles reveals insight into metal deposition processes. *ChemCatChem* **7**, 3674–3682 (2015).

18. F. Meirer, S. Kalirai, J. N. Weker, Y. Liu, J. C. Andrews, B. M. Weckhuysen, Agglutination of single catalyst particles during fluid catalytic cracking as observed by X-ray nanotomography. *Chem. Commun.* **51**, 8097–8100 (2015).

19. A. M. Wise, J. N. Weker, S. Kalirai, M. Farmand, D. A. Shapiro, F. Meirer, B. M. Weckhuysen, Nanoscale chemical imaging of an individual catalyst particle with soft X-ray ptychography. *ACS Catal.* **6**, 2178–2181 (2016).

20. S. De Meyer, F. Vanmeert, R. Vertongen, A. van Loon, V. Gonzalez, G. van der Snickt, A. Vandivere, K. Janssens, Imaging secondary reaction products at the surface of vermeer's girl with the pearl earring by means of macroscopic X-ray powder diffraction scanning. *Herit. Sci.* **7**, 67 (2019).

21. L. Monico, L. Cartechini, F. Rosi, A. Chieli, C. Grazia, S. De Meyer, G. Nuyts, F. Vanmeert, K. Janssens, M. Cotte, W. De Nolf, G. Falkenberg, I. Crina, A. Sandu, E. Storevik Tveit, J. Mass, R. Pereira De Freitas, A. Romani, C. Miliani, Probing the chemistry of CdS paints in The Screamby in situ noninvasive spectroscopies and synchrotron radiation x-ray techniques *Sci. Adv.* **6**, eaay3514(2020).

22. K. Groen, E. van de Wetering, Grounds in Rembrandt's workshop and in paintings by his contemporaries, in *A Corpus of Rembrandt Paintings IV*, Stichting Foundation Rembrandt Research Project book series (Springer, 2005), pp. 318–334.

23. K. Groen, Earth Matters. The origin of the material used for the preparation of the Night Watch and many other canvases, in Rembrandt's Workshop After 1640 (ARTMATTERS - Netherlands Technical Studies in Art, 2005), pp. 138–154.

24. J. Dik, K. Janssens, G. V. D. Snickt, L. V. D. Loeff, K. Rickers, M. Cotte, Visualization of a lost painting by vincent van gogh using synchrotron radiation based X-ray fluorescence elemental mapping. *Anal. Chem.* **80**, 6436–6442 (2008).

25. M. Alfeld, J. V. Pedroso, M. van Eikema Hommes, G. Van der Snickt, G. Tauber, J. Blaas, M. Haschke, K. Erler, J. Dik, K. Janssens, A mobile instrument for in situ scanning macro-XRF investigation of historical paintings. *J. Anal. At. Spectrom.* **28**, 760–767 (2013).

26. J. K. Delaney, K. A. Dooley, A. van Loon, A. Vandivere, Mapping the pigment distribution of vermeer's girl with a pearl earring. *Herit. Sci.* **8** (2020).

27. F. Gabrieli, J. K. Delaney, R. G. Erdmann, V. Gonzalez, A. van Loon, P. Smulders, R. Berkeveld, R. V. Langh, K. Keune, Reflectance imaging spectroscopy (RIS) for operation night watch: Challenges and achievements of imaging Rembrandt's masterpiece in the glass chamber at the rijksmuseum. *Sensors* **21**, 6855 (2021).

28. T. Missana, M. Garcia-Gutierrez, U. Alonso, Sorption of strontium onto illite/smectite mixed clays. *Phys. Chem. Earth* **33**, S156–S162 (2008).

29. J. I. Goldstein, D. E. Newbury, J. R. Michael, N. W. M. Ritchie, J. H. J. Scott, D. C. Joy, *Scanning Electron Microscopy and X-Ray Microanalysis* (Springer, 2017).

30. L. Baij, J. Hermans, B. Ormsby, P. Noble, P. Iedema, K. Keune, A review of solvent action on oil paint. *Herit Sci* **8**, 43 (2020).

31. M. Cotte, E. Checroun, W. De Nolf, Y. Taniguchi, L. De Viguerie, M. Burghammer, P. Walter, C. Rivard, M. Salomé, K. Janssens, J. Susini, Lead soaps in paintings: Friends or foes? *Stud. Conserv.* **62**, 2–23 (2017).

32. T. T. De Mayerne, *Sloane MS 2052 Pictoria, Sculptoria, Tinctoria et quae subalternarum Artium* (British Library, 1620-1646).

33. G. Heydenreich, *Lucas Cranach, the Elder. Painting Materials Techniques and Workshop Practice* (Amsterdam Univ. Press, 2007).

34. P. J. J. van Thiel, Damaging and restoration night watch. *Bulletin van het Rijksmuseum* **24**, 4–13 (1976).

35. L. Kuiper, W. Hesterman, Report on the restoration of Rembrandt's Night Watch. *Bulletin van het Rijksmuseum* **24**, 14–51 (1976).

36. G. Van de Voorde, The radiography of Rembrandt's Night Watch. *Bulletin van het Rijksmuseum* **24**, 52–67 (1976).

37. E. van de Wetering, C. M. Groen, J. A. Mosk, Summary report on the results of the technical examination of Rembrandt's Night Watch. *Bulletin van het Rijksmuseum* **24**, 68–98 (1976).

38. F. Casadio, K. Keune, P. Noble, A. Van Loon, E. Hendriks, S. A. Centeno, G. Osmond, *Metal Soaps in Art: Conservation and Research* (Springer International Publishing, 2019).

39. J. J. Hermans, K. Keune, A. van Loon, P. D. Iedema, The crystallization of metal soaps and fatty acids in oil paint model systems. *Phys. Chem. Chem. Phys.* **18**, 10896–10905 (2016).

40. J. J. Boon, J. van der Weerd, K. Keune, P. Noble, J. Wadum, Mechanical and chemical changes in old master paintings: Dissolution, metal soap formation and remineralization processes in lead pigmented ground/intermediate paint layers of 17th century paintings, in *ICOM Committee for Conservation, 13th Triennial Meeting*, Rio de Janeiro, 22–27 September 2002, vol. II, pp. 401–406.

41. K. Keune, J. J. Boon, Analytical imaging studies of cross-sections of paintings affected by lead soap aggregate formation. *Stud. Conserv.* **52**, 161–176 (2007).

42. J. J. Hermans, K. Keune, A. van Loon, R. W. Corkery, P. D. Iedema, Ionomer-like structure in mature oil paint binding media. *RSC Adv.* **6**, 93363–93369 (2016).

43. J. J. Hermans, A. van Loon, K. Keune, P. D. Iedema, *Toward a Complete Molecular Model for the Formation of Metal Soaps in Oil Paints in Metal Soaps in Art: Conservation and Research*, F. Casadio, K. Keune, P. Noble, A. van Loon, E. Hendriks, S. A. Centeno, G. Osmond, Eds. (Springer International Publishing, 2019).

44. C. Higgitt, M. Spring, D. Saunders, Pigment-medium Interactions in oil paint films containing red lead or lead-tin yellow. *NAACOG Tech. Bull.* **24** (2003).

45. S. A. Centeno, D. Mahon, The chemistry of aging in oil paintings: Metal soaps and visual changes. *MMAB* **67**, 12–19 (2009).

46. Y. K. Chen-Wiegart, J. Catalano, G. J. Williams, A. Murphy, Y. Yao, N. Zumbulyadis, S. A. Centeno, C. Dybowski, J. Thieme, Elemental and molecular segregation in oil paintings due to lead soap degradation. *Sci. Rep.* **7**, 11656 (2017).

47. P. Noble, A brief history of metal soaps in paintings from a conservation perspective, in *Metal Soaps in Art: Conservation and Research*, F. Casadio, K. Keune, P. Noble, A. Van Loon, E. Hendriks, S. A. Centeno, G. Osmond, Eds. (Springer International Publishing, 2019).

48. F. C. Izzo, M. Kratter, A. Nevin, E. Zendri, A critical review on the analysis of metal soaps in oil paintings. *ChemistryOpen* **10**, 904–921 (2021).

49. V. Gonzalez, I. Fazlic, M. Cotte, F. Vanmeert, A. Gestels, S. De Meyer, F. Broers, J. Hermans, A. van Loon, K. Janssens, P. Noble, K. Keune, Lead(II) formate in Rembrandt's Night Watch: Detection and distribution from the macro- to the micro-scale. *Angew. Chem. Int. Ed.* **62**, e202216478 (2023).

50. E. van Duijn, J. P. F. Kok, The restorations of Rembrandt's Night Watch. *Burlingt. Mag.* **158**, 117–128 (2016).

51. K.-J. Kim, T.-J. Eom, Chemical characteristics of degraded beeswax in the waxed volume of the annals of King Sejong in the Joseon Dynasty. *J. Cult. Herit.* **16**, 918–921 (2015).

52. J. Mazurek, M. Svoboda, M. Schilling, GC/MS characterization of beeswax, protein, gum, resin, and oil in romano-egyptian paintings. *Herit.* **2**, 1960–1985 (2019).

53. E. van Duijn, M. Te Marvelde, Hopman and de wild: The historical importance of two dutch families of restorers. *Burlingt. Mag.* **158**, 812–823 (2016).

54. A. van Loon, K. Keune, J. J. Boon, Improving the surface quality of paint cross-sections for imaging analytical studies with specular reflection FTIR and Static-SIMS, in *Art'05 - 8th International Conference on Non Destructive Investigations and Micronalysis for the Diagnostics and Conservation of the Cultural and Environmental Heritage*, Lecce, Italy, 15–19 May 2005 (Italian Society of Non Destructive Testing Monitoring Diagnostics).

55. V. A. Solé, E. Papillon, M. Cotte, Ph. Walter, J. Susini, A multiplatform code for the analysis of energy-dispersive X-ray fluorescence spectra. *Spectrochim. Acta B At. Spectrosc.* **62**, 63–68 (2007).

56. A. M. Maiden, J. M. Rodenburg, An improved ptychographical phase retrieval algorithm for diffractive imaging. *Ultramicroscopy* **109**, 1256–1262 (2009).

57. Y. Liu, F. Meirer, P. A. Williams, J. Wang, J. C. Andrews, P. Pianetta, TXM-wizard: A program for advanced data collection and evaluation in full-field transmission X-ray microscopy. *J. Synchrotron Radiat.* **19**, 281–287 (2012).

58. Operation Night Watch; www.rijksmuseum.nl/en/whats-on/exhibitions/operation-night-watch.
